# Real-world genomic profiling of solid tumors: validation and clinical insights from a Brazilian cohort

**DOI:** 10.31744/einstein_journal/2026AO2000

**Published:** 2026-06-08

**Authors:** Thalita Xavier de Souza, Roberta Cardoso Petroni, Larissa Barbosa de Lima, Luiz Gustavo Ferreira Cortes, Gustavo Santos de Oliveira, Caroline Nunes Silveira, Nair Hideko Muto, Joice Rosa Santana, Priscila Iamashita Higaki, Amanda Centenaro Ramos, Susana Elaine Alves da Rosa, Rodrigo Reis, Miguel Zugman, Fernando Moura, Pedro Luiz Serrano Usón, Paulo Vidal Campregher

**Affiliations:** 1 Hospital Israelita Albert Einstein São Paulo SP Brazil Hospital Israelita Albert Einstein, São Paulo, SP, Brazil.; 2 Genesis Genomics São Paulo SP Brazil Genesis Genomics, São Paulo, SP, Brazil.

**Keywords:** Precision medicine, Neoplasms, High-throughput nucleotide sequencing, Genomic structural variation, Tumor molecular profiling, Illumina TruSight Oncology 500, Somatic alterations, Brazilian molecular data

## Abstract

**Background:**

Precision medicine has transformed cancer management by tailoring treatments to the molecular characteristics of a patient’s tumor. Comprehensive Genomic Profiling enables the simultaneous analysis of multiple genomic alterations using next-generation sequencing assays, providing detailed molecular profiles of tumors. This approach allows more accurate cancer classification, guides decisions regarding targeted therapies, enhances the precision of treatment strategies, and improves patient outcomes in oncology.

**Objective:**

In this article, we describe the analytical validation of the Illumina TruSight Oncology 500 (TSO500) assay in a (CAP)-certified clinical laboratory in Brazil and present real-world findings from Comprehensive Genomic Profiling performed on 454 patients with cancer, highlighting the genomic landscape, including novel fusion events.

**Methods:**

Tumor samples collected between January 2020 and September 2023 were subjected to Comprehensive Genomic Profiling using the TSO500 assay. Library preparation and sequencing (Illumina NextSeq) were performed according to the manufacturer’s protocol, followed by bioinformatics processing using an in-house pipeline for alignment, variant calling, and annotation. Variants were classified according to the Variant Interpretation for Cancer Consortium guidelines. Analytical performance metrics, including specificity and positive predictive value, were calculated for assay validation.

**Results:**

The test demonstrated a specificity of 93.39% and a positive predictive value of 73.36%. Regarding Comprehensive Genomic Profiling, 57.85% of all detected variants were classified as variants of uncertain significance, and 42.15% were oncogenic. Gene fusions, including both novel and canonical events, were detected in 13% of cases.

**Conclusion:**

The TSO500 assay provides crucial insights into patient genomics, aiding diagnosis, prognosis, and treatment decisions. Demonstrating high levels of accuracy, reproducibility, and sensitivity, the TSO500 advances cancer research to the forefront of molecular technology.

## Highlights

■ TSO500 showed 93.39% sensitivity and high concordance with FoundationOne.

■ Comprehensive Genomic Profiling of 454 tumors revealed the genomic landscape of 57 cancer types.

■ Variants of uncertain significance comprised 57.9% of all genomic events, highlighting challenges in precision oncology.

■ Gene fusions occurred in 13% of cases, with 45.5% classified as variants of uncertain significance.

## In Brief

We validated the TSO500 assay in a CAP-accredited Brazilian laboratory and analyzed the Comprehensive Genomic Profiling results from 454 patients with cancer. The TSO500 assay showed high analytical performance and revealed key genomic alterations, including novel fusions, supporting precision-guided cancer management.

## Graphical abstract

**Figure:**
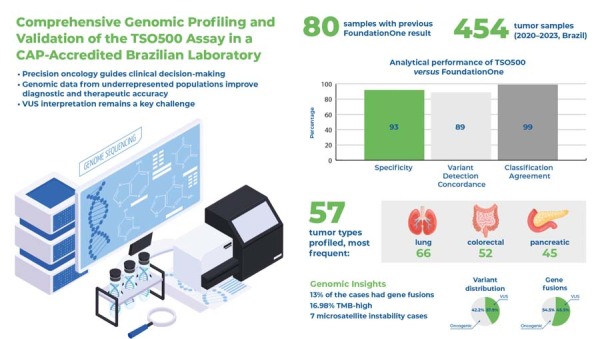

## INTRODUCTION

Cancer is caused by the progressive accumulation of oncogenic genetic abnormalities and is the second leading cause of mortality worldwide. One of the main factors contributing to this high mortality rate is the frequently late diagnosis, which in most cases occurs when the disease is already at advanced stages, limiting the available therapeutic options.^(1)^ Therefore, there has been significant effort from the scientific community to develop new diagnostic and treatment strategies aimed at improving the quality of life and longevity of patients, especially those with cancer. Recently, significant advancements have been made in genomic approaches to cancer, owing to the identification of molecular alterations that can be therapeutically targeted, aiding in the clinical management of this disease.

Next-generation sequencing (NGS) has become an essential tool in clinical oncology. It serves as the foundation for precision medicine applications because it can sequence the entire genome or only specific areas of interest. Identifying clinically actionable molecular alterations is an important part of cancer diagnosis and the selection of appropriate treatment strategies.^(2)^ The use of NGS in clinical practice enables treatment response prediction, improves prognostic definition, identifies resistance mechanisms, and helps germline predisposition syndromes.

The premise of Precision Medicine in oncology is that treatment choices based on the unique and individual characteristics of the patient’s disease, after thorough investigation of its molecular base, will lead to better outcomes. This deepens our understanding of tumor genomic profiles, enabling personalized treatment strategies that may be more effective and less toxic than conventional treatments for cancer.

Based on that, the use of NGS for Comprehensive Genomic Profiling (CGP) for solid tumors increases the chances of identifying clinically relevant alterations^(3)^ potentially actionable with targeted therapies. The molecular alterations evaluated in a CGP include single nucleotide polymorphisms (point mutations), structural gene rearrangements such as translocations, fusions, insertions, and deletions, copy number variations, as well as relevant biomarkers such as tumor mutational burden (TMB) and microsatellite instability (MSI).^(4)^

Our goal was to validate TruSight Oncology 500 (TSO500), an NGS panel widely used for CGP, in an College of American Pathologists (CAP)-certified clinical laboratory, and to describe our experience with CGP in clinical practice, focusing on novel findings and challenges for the classification and curation of diverse genomic alterations. Our findings contribute to the accumulation of real-world data, which is essential to demonstrate the viability of precision medicine, and will contribute to generating evidence of the effectiveness of this strategy.^(5)^

## OBJECTIVE

In this study, we aimed to validate the TSO500 next-generation sequencing panel in a (CAP)-certified clinical laboratory and describe our real-world experience with Comprehensive Genomic Profiling, emphasizing novel findings and challenges in the classification and curation of diverse genomic alterations.

## METHODS

### Ethical approval

This study was approved by *Hospital Israelita Albert Einstein* (HIAE) Institutional Review Board CAAE: 72996523.0.0000.0071; # 6.541.648. Informed consent was obtained from all patients who provided authorization for the use of their molecular data through an online consent form.

### Sample sequencing

**Figure 1:**
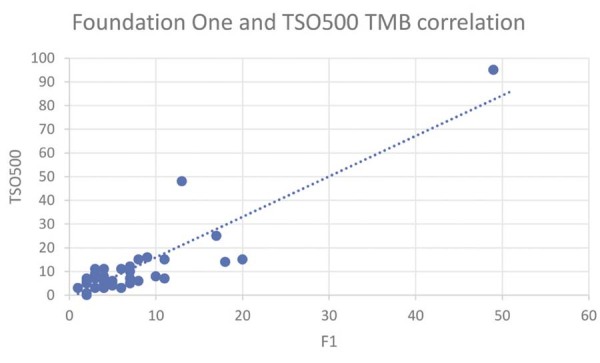
Correlation of tumor mutational burden performance between the TSO500 and FoundationOne assays

To validate the TSO500 system (Illumina, San Diego, CA, USA), we tested 80 samples for accuracy, two samples of DNA and RNA for reproducibility, and one sample in multiple runs to assess the limit of detection. Specifically, 46 DNA and 34 RNA samples were selected based on the criteria outlined in Figure 1S, Supplementary Material. For CGP and analysis of real-world data, 454 patients were included. Different tumor types containing at least 20% of the tumoral area in the histological slides were analyzed.

Nucleic acid extraction was performed using All Prep (QIAGEN, Hilden, Germany), QiaAmp FFPE (QIAGEN), or ReliaPrep (Promega, Madison, WI, USA) kits, and the samples were stored at -80°C until use. After nucleic acid quantification, quality was evaluated using Tapestation (Agilent Technologies, Santa Clara, CA, USA) equipment with the High Sensitivity D1000 Screen Tape and RNA Screen Tape reagents for DNA and RNA, respectively.

Libraries were prepared according to the TruSight Oncology 500 Reference Guide. First, the DNA samples were fragmented using the ME220 system (Covaris, Woburn, MA, USA). In summary, fragmented DNA and cDNA from RNA samples were indexed and underwent two cycles of hybridization and target region capture. After normalization, the samples were quantified and pooled. Sequencing was then performed using the Illumina NextSeq platform, and the resulting data were processed using the bioinformatics pipeline outlined below.

### Bioinformatics pipeline

An in-house pipeline was developed based on the default configuration outcomes of the TruSight Oncology (TSO) pipeline v2.2.0 for downstream analysis. For single nucleotide polymorphism (SNP) and insertion-deletion (indel) analyses, stitched realigned BAM files were used as inputs for SNP calling with Freebayes v1.0.2 and Mutect2 from GATK v4.1.5.0, employing a minimum base quality score of 30 and a callable depth of 3. Variants identified by Pisces (the TSO standard variant caller), Freebayes, and Mutect2 were merged and annotated using ANNOVAR (version 2019Oct24). The annotated files were filtered based on a predefined list of target genes.

To assess the presence of MSI, the TSO pipeline evaluates 130 repeat loci;^(6)^ if a minimum of 40 microsatellites were evaluable, MSI analysis was performed using PercentageUnstableSites. Microsatellite instability was considered positive if at least 20% of the microsatellites were unstable. For TMB, if the value of adjusted TmbPerMb was >10 mutations/Mb, TMB was considered high (TMB-H).

Copy number variants (CNVs) were analyzed from the output of the TSO application. First, the VCF files were annotated using AnnotSV v2.3.2, and the variants were filtered based on a targeted gene list for further evaluation. To complement this analysis, particularly for deletion events in the *CDKN2A/B* genes, a pipeline based on the GATK4 somatic workflow v1.4.0 was implemented. A negative control tumor panel (NCTP) comprising 83 tumor samples with minimal CNVs was established to validate the detection of *CDKN2A/B* deletions.

For RNA analysis, fusions were evaluated using a combined approach that included the VCF output from the TSO pipeline and Arriba v.2.3.0. A list of hotspot fusions was used to filter and highlight the relevant clinical events. RNA splicing variant outputs in the VCF files from the TSO pipeline were also annotated using ANNOVAR and filtered based on the same list of targeted genes used for CNV analysis.

### Variant analysis

The TSO500 assay identifies 523 genes and 55 RNA transcripts. For clinical purposes, two reporting gene sets were used: an initial panel of 159 genes (v1) and an expanded panel implemented in June 2023 (v2), which retained all v1 genes and included other clinically relevant targets, totaling 351 genes (Table 1S, Supplementary Material), such as those with diagnostic, prognostic, and therapeutic potential, and genes associated with hereditary cancer predisposition syndromes.

For artifact exclusion, we developed a metric that evaluated the occurrence of each variant among all sequenced samples and the allele frequency distribution of the previously detected variant. Variants frequently detected in the historic cohort within the allele frequency range typical of artifacts were excluded. Cancer hotspots were not included in this filtering and were always flagged for detailed evaluation. Variants considered real with an allele frequency above 5% were included in the analysis.

**Figure 2:**
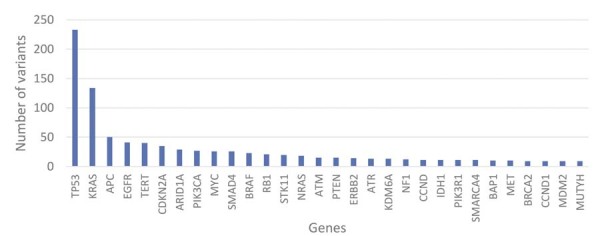
The 30 most frequently mutated genes in the 454 included cases, with oncogenic variants

Variant classification was performed based on the Variant Interpretation for Cancer Consortium (VICC) criteria.^(7)^ Variants were classified as benign, likely benign, variants of uncertain significance (VUS), likely oncogenic, or oncogenic. Specialized literature and public databases, including GNOMAD, ClinVar, Franklin, and cBioPortal were used for variant classification. Gene fusions were analyzed using the IGV program. If both genes had at least 20 supporting reads, including split reads and unmatched pairs, the fusion was considered true. Figure 2S, Supplementary Material shows a diagram of the TSO500 workflow from sample extraction to results reporting.

### Statistical analysis

Statistical analyses were performed to evaluate the technical validation of the TSO500 and the molecular characterization of the Brazilian cohort of patients with solid tumors.

The performance metrics for the analytical validation were calculated using the FoundationOne test as a reference. Variant concordance between the TSO500 panel and F1 test was assessed using the percentage of shared variants.

The frequencies of genomic alterations were calculated according to variant type (SNVs, indels, gene fusions, and CNVs) and the affected genes. The proportion of variants classified as oncogenic, likely oncogenic, or VUS was determined. For the TMB and MSI analyses, measures of central tendency (mean, median), dispersion (standard deviation), and proportion of high-level cases (*e.g*., TMB ≥10 mut/Mb) were calculated. Data distributions were evaluated across tumor types to explore potential associations between genomic burden and histological subtypes.

## RESULTS

### Validation of the TSO500 molecular panel (Illumina)

The TSO500 test (Illumina) was validated in our laboratory following the CAP guidelines. The validation process included accuracy, intra- and inter-assay reproducibility, and definition of limit of detection. The evaluated parameters included DNA variant detection (SNV and Indels), TMB, MSI, CNVs, and gene fusion detection (RNA-based). The panel was validated using paraffin-embedded tissue samples obtained from different sites (Table 2S, Supplementary Material).

Quality control was performed by evaluating the minimum number of target reads, average fragment size, median absolute deviation for CNVs, uniformity, usable sites for MSI, contamination score, and average read coverage according to the manufacturer’s recommendations. Additionally, bioinformatics analyses were performed to validate the pipeline developed by the laboratory using the VarStation platform.

### Accuracy of the assay

To assess the accuracy of somatic variant identification, TMB, and MSI, we used 46 DNA samples and compared their results with those of the FoundationOne test, which is considered the gold standard.

With the TSO500 assay, we identified a total of 305 DNA variants. Among them, 212 variants were detected by FoundationOne, resulting in an overall concordance of 88.70%. Regarding discrepancies, 81 variants detected by TSO500 were not reported by FoundationOne: 4 were in genes not covered by the FoundationOne panel (*ERCC2* and *PHOX2B*), whereas 77 were confirmed upon IGV inspection. In contrast, 15 variants reported by FoundationOne were not detected by TSO500, primarily owing to their variant allele frequency (VAF) being below 5%, high population frequency, or absence in IGV inspection. Additionally, 11 variants detected by both assays were classified as benign by our team, and 1 variant was considered an artifact.

Performance analysis of the TSO500 assay revealed a sensitivity of 93.39%, indicating a good ability to detect variants identified by the FoundationOne assay. Additionally, the positive predictive value was calculated to be 73.36%, reflecting the proportion of true positive variants among all those reported as positive by the TSO500.

To classify the 212 variants detected by both assays, we compared the designation of the variants as either VUS or oncogenic/probably oncogenic. Among these, we observed three discrepancies: one variant classified as oncogenic by FoundationOne was classified as a VUS by our team (NM_015125.4:c.310G>A, p.Glu104Lys), whereas two variants classified as VUS by FoundationOne were categorized as oncogenic/probably oncogenic by our team (NM_000455.4:c.526G>A, p.Asp176Asn and NM_020975.6:c.2410G>T, p.Val804Leu). This resulted in 98.60% correlation between the two assays for variant classification.

Tumor mutational burden accuracy was assessed in two ways: as a continuous variable compared with correlation among all cases, resulting in a correlation coefficient (R) of 0.91, and when using the FDA-recognized cutoff of 10 mutations per megabase (mut/Mb). The accuracy of classifying cases above or below the cutoff was 86% compared to that of FoundationOne (R=0.86) (Figure 1).

For the fusion analysis, 34 RNA samples were used and compared with previous results obtained from FoundationOne CDx (Foundation Medicine) or Oncomine (ThermoFisher). Additionally, a commercial control, SeraSeq RNA Fusion (SeraCare), was used to assess the accuracy of fusion detection. The correlation achieved for fusion detection was 96%.

To evaluate gene amplification (CNVs), 46 samples with previous results (20 positive and 26 negative) from the FoundationOne CDx Test (Foundation Medicine) were used. Amplifications in 18 samples were confirmed using TSO500, resulting in a sensitivity of 92.30%. A specificity of 99.80% was achieved when we used a copy number cutoff ≥6. If the values of the fold change were ≥2.1 and <2.3 and the copy numbers were ≥5.1 and <6, amplification was considered suspicious.

To validate deletion detection with TSO500, we focused on the genes *CDKN2A* and *CDKN2B* as they are located in close proximity, and deletions in this region frequently span several hundred nucleotides. Two groups of samples were used: an NCTP consisting of 83 tumor samples (TC > 60%) with no CNVs detected in prior TSO500 runs, and 48 samples used for accuracy assessment, which already had a previous result using FoundationOne, of which 39 were negative and 9 were positive for homozygous deletions in *CDKN2A/B*. The overall accuracy of TSO500 was 93.70%, with 3 out of the 48 samples showing discordant results with FoundationOne (1 ≤ copy number ≤ 1.35 or 0.7 ≤ copy ratio ≤ 0.85).

### Reproducibility and limit of detection

To assess the reproducibility of the TSO500 test, two DNA samples and two RNA samples were tested in five inter- and intra-assay replicates. We obtained 100% reproducibility for all replicates.

To establish the analytical sensitivity of the assay, we assessed its limit of detection (LoD) using a sample containing an *MEN1* variant (NM_000244: exon10:c.1534G>A, p.G512S) with a VAF of 5%. This variant was reliably detected across all three replicates, with VAFs ranging from 0.058 to 0.09, confirming an LoD of 5%. This consistency in detection underscores the sensitivity of the test at lower allele frequencies, which is critical for the accurate identification of variants present at low levels in heterogeneous samples.

### Variants detected in clinical samples

Comprehensive Genomic Profiling was performed on 454 samples using TSO500. Although the TSO500 assay assessed a total of 578 genes, we focused our gene analysis on 250 genes considered clinically relevant. Oncogenic/likely oncogenic mutations were identified in 131 genes and VUS in 148. The 30 genes with the highest number of oncogenic variants were *TP53*, *KRAS*, *APC*, and *EGFR* (Figure 2).

**Figure 3:**
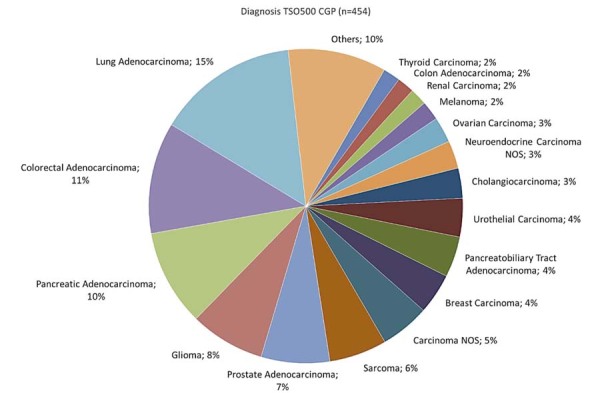
Most frequent types of tumors found in the 454 cases analyzed

Among the 454 patients with CGP results, 37 did not present with any oncogenic or likely oncogenic variants (Table 3S, Supplementary Material), However, in the 417 positive cases, we identified 1,188 oncogenic alteration events and 1,630 VUS across all variant types, including SNVs, indels, fusions, and amplifications. Overall, 57.85% of the detected variants were VUS, and 42.15% were oncogenic (Table 4S, Supplementary Material), with an average of 2.61 oncogenic variants and 3.10 VUS per case. Analysis of the distribution of variant types among cases showed that the most common variants were SNVs, followed by indels, CNV, and gene fusions (Figure 3S, Supplementary Material).

### Diagnosis

We profiled 57 different tumor types, the most common being lung (66 cases), colorectal (52 cases), and pancreatic adenocarcinoma (45 cases). Among the total of 57 tumor types, 40 less frequent types were represented in figure 3 as “Others,” as they each occurred in only 1 to 7 cases.

### Tumor mutational burden and MSI determination

The average TMB was 7.47 mut/Mb, ranging from 0.78 to 85.6 mut/Mb. TMB was reported in 424 cases, with 72 cases presenting levels above 10 mut/Mb, indicating that 16.98% of the analyzed cases were TMB-H.

**Figure 4:**
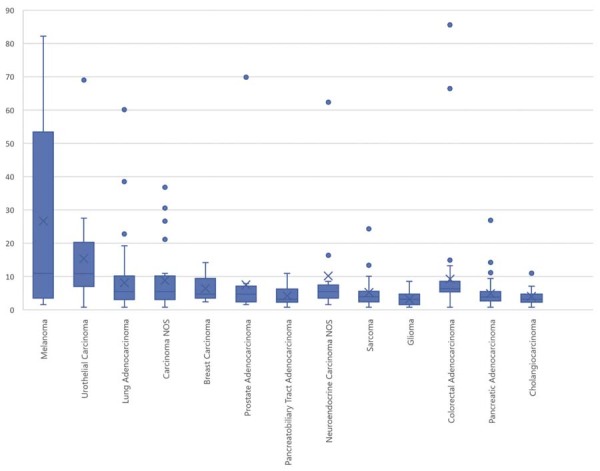
Tumor mutational burden variation in the Comprehensive Genomic Profiling analysis

Figure 4 illustrates the 13 tumor types that had a significant representation of TMB cases, with melanoma showing the greatest variation. The median TMB varied significantly among tumor types, a common phenomenon that plays a crucial role in influencing the potential response to immune checkpoint inhibitors.^(8)^

The presence of numerous outliers in tumors such as sarcoma, lung cancer, and colorectal adenocarcinoma suggests that there are individual cases within these tumor types with exceptionally high TMB, which could be particularly relevant for therapeutic decisions.

**Figure 5:**
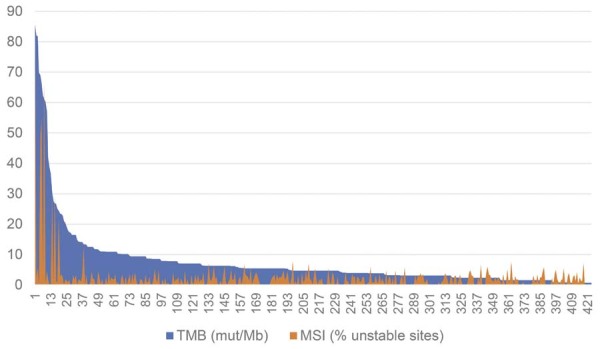
Tumor mutational burden and microsatellite instability (MSI) correlation (TMB>0)

Regarding microsatellite analysis, only seven cases exhibited MSI, defined as having 20% or more unstable sites; all these MSI cases also exhibited TMB-H. The median TMB was 61.3 and 4.7 mut/Mb in the MSI and microsatellite stability (MSS) cases, respectively. The correlation between TMB and MSI status in 424 patients with TMB>0 is shown in figure 5. Interestingly, all cases with MSS had <10% unstable microsatellites, with one exception: a case of a soft tissue sarcoma with 12.63% unstable microsatellites and 13.4 mut/Mb, which was classified as MSS. Nevertheless, this patient had immunohistochemistry results showing a loss of nuclear expression of the DNA repair system enzymes MLH-1 and PMS2, and had a clinical response to immunotherapy.^(9)^

### Gene fusions

A total of 66 gene fusions were identified in 59 of the 454 cases, representing 13% of the samples analyzed. Among them, 55 were unique. Of these, 25 were classified as VUS and 30 were deemed oncogenic (Figure 4S, Supplementary Material). Nineteen of the oncogenic fusions were canonical, as they are well documented and characterized in the scientific literature, as represented in orange in Figure 4S, Supplementary Material and detailed in table 1.

**Table 1:** Canonical oncogenic fusions with the corresponding tumor type

Canonical oncogenic fusions	
SLC45A3::ERG	Prostate adenocarcinoma
TMPRSS2::ERG	Prostate adenocarcinoma (8)
SND1::BRAF	Prostate adenocarcinoma
TMPRSS2::ETV4	Prostate adenocarcinoma
KIF5B:: RET	Lung adenocarcinoma
CD74::ROS1	Lung adenocarcinoma
CD74:: NRG1	Lung adenocarcinoma
ALK::EML4	Lung adenocarcinoma
SDC4::ROS1	Lung adenocarcinoma
MET (exon 14 skipping)	Lung adenocarcinoma (2)
KIAA1549::BRAF	Glioma (5)
FGFR2::SHTN1	Glioma
SEC 61G::EGFR	Glioma
EWSR1::WT1	Sarcoma
SCD5::ALK	Sarcoma
LMNA::NTRK1	Sarcoma
PAX3::FOXO1	Sarcoma
PAX3::NCOA2	Sarcoma
GOPC::ROS1	Colorectal adenocarcinoma

## DISCUSSION

In this article, we present a validation process for the TSO500 assay in a (CAP)-certified clinical laboratory. This NGS assay enables more accurate diagnoses and the provision of specific treatments based on the molecular findings for each patient obtained through CGP, assessing 523 cancer-related genes from DNA samples and 55 genes from RNA samples, providing a comprehensive molecular profile of tumors.

When analyzing the results of the TSO accuracy validation, we observed some discrepancies in the detection and classification of somatic variants. However, these differences seemed to stem primarily from the high sensitivity of the TSO assay rather than fundamental disagreements in variant classification. The lack of universal consensus on variant classification criteria also plays a role, as each laboratory may adopt distinct methodologies. In the HIAE laboratory, we followed the VICC guidelines;^(7)^ however, variability in interpretation across institutions reinforces the need for standardized approaches. Additionally, limited sample sizes for certain populations impact the classification of VUS, highlighting the importance of continued research and larger datasets for refining these classifications.

After validation, the TSO500 assay showed high precision, accuracy, sensitivity, and reproducibility, and was incorporated into routine laboratory practice at the HIAE. Since then, we have analyzed data from 454 patients with 57 different tumor types. In the CGP analysis reported in this study, we focused on 250 actionable genes because the TSO500 assesses genes that are directly linked to cancer. This selection is critical for prioritizing relevant findings for therapeutic decisions.

Our findings revealed that 42.15% of the identified variants were oncogenic, whereas 57.85% were VUS. The detected variants included SNVs, indels, fusions, and CNVs, highlighting the ability of this assay to detect diverse mutations. Many of these variants have been extensively studied, such as NM_004333.6:c.1799T>A and p.Val600Glu, which are highly recurrent in melanoma, lung, and thyroid cancers^(10)^ and represent actionable alterations with potential clinical implications. However, the high prevalence of VUS in our study reflects the ongoing challenge of interpreting genomic data and the need for continued functional studies and database curation. These findings emphasize the utility of genomic profiling for precision oncology and the importance of addressing areas of ambiguity to enhance clinical decision-making. Research on VUS classification could reveal new biomarkers for precision oncology, helping to refine diagnosis and treatment approaches.^(11)^

Gene fusion events are prevalent in prostate cancer, gliomas, and lung adenocarcinomas. Among the 11 prostate cancer cases in this study showing gene fusions, eight exhibited a TMPRSS2::ERG fusion, a well-recognized biomarker in prostate adenocarcinoma.^(12)^ Furthermore, 25 of the 55 fusions identified in this CGP analysis were classified as VUS, highlighting the complexity of interpreting fusion events in clinical contexts. These findings underscore the need for more functional studies to determine the roles of these variants in cancer progression and therapeutic responses.^(11)^

Tumor mutational burden and MSI have emerged as critical biomarkers for identifying patients who may benefit from immunotherapy.^(13)^ In this study, 16.98% of cases presented TMB levels above 10 mut/Mb, a threshold considered high by the FDA, while MSI-H was observed in 7 cases. All MSI-H cases were accompanied by a high TMB, reinforcing the clinical significance of these biomarkers in guiding cancer treatments. A notable case of a soft tissue sarcoma with 12.63% unstable microsatellites, classified as MSS, highlights the limitations of MSI testing with NGS alone. Despite the MSS classification, immunohistochemistry (IHC) revealed the loss of a key protein involved in mismatch repair (MMR), explaining the positive response to immunotherapy.^(9)^ This case underscores the importance of combining MSI and IHC testing to identify potential MMR deficiencies that could affect treatment outcomes.^(8)^

This study has some limitations, including the lack of complete clinical data, such as tumor stage, treatment, and patient outcomes, which limited further analyses. There is also a significant gap in knowledge regarding variants of uncertain significance (VUS), especially in underrepresented populations such as the Brazilian population. Future studies should focus on integrating clinical data to better understand the clinical relevance of the detected alterations and on performing functional studies to clarify the role of VUS.

## CONCLUSION

Here, we validated the performance of the TSO500 assay and demonstrated its high reliability, accuracy, sensitivity, and reproducibility in clinical laboratory settings. Comprehensive Genomic Profiling of 454 patients with solid tumors revealed a broad spectrum of somatic alterations, with 42.15% classified as oncogenic and 57.85% as variants of uncertain significance, underscoring the ongoing need for functional studies and database expansion to improve variant interpretation.

Tumor mutational burden and microsatellite instability have emerged as relevant biomarkers for immunotherapy guidance; however, their clinical use should be integrated with complementary assays, such as immunohistochemistry, in specific contexts. By generating real-world molecular data from a historically underrepresented population, this study contributes to the growing effort to make precision oncology more inclusive and population-specific.

Moreover, the successful validation of the TSO500 in this setting provides a replicable framework for implementing Comprehensive Genomic Profiling in diverse clinical environments, supporting its integration into routine oncology practice and the potential to reveal actionable patterns through high-throughput sequencing technologies.

## SUPPLEMENTARY MATERIAL

SUPPLEMENTARY MATERIAL
