# Supplementary material for: Real-world genomic profiling of solid tumors: validation and clinical insights from a Brazilian cohort
Source: Einstein (Sao Paulo). 2026 Jun 8;24:eAO2000. doi: 10.31744/einstein_journal/2026AO2000 (PMC13399300; doi:10.31744/einstein_journal/2026AO2000)
Supplement: SUPPLEMENTARY MATERIAL [file 2317-6385-eins-24-eAO2000-suppl01.pdf]

SUPPLEMENTARY MATERIAL

Real-world genomic profiling of solid tumors: validation and clinical insights from a Brazilian cohort

Thalita Xavier de Souza, Roberta Cardoso Petroni, Larissa Barbosa de Lima, Luiz Gustavo Ferreira Cortes, Gustavo Santos de Oliveira, Caroline Nunes Silveira, Nair Hideko Muto, Joice Rosa Santana, Priscila Iamashita Higaki, Amanda Centenaro Ramos, Susana Elaine Alves da Rosa, Rodrigo Reis, Miguel Zugman, Fernando Moura, Pedro Luiz Serrano Usón Junior, and Paulo Vidal Campregher

DOI: 10.31744/einstein_journal/2026AO2000

Table 1S. Genes selected for variant reporting. Genes were initially reported using version 1 (v1, 159 genes) and, from June 2023 onwards, with version 2 (v2, 351 genes, including all genes in v1 plus additional clinically relevant targets)

Genes selected for variant reporting	DNA	Fusion	Amplification	Version
ABL1		✓		v1
ACVR1	✓			v2
ACVR1B	✓			v2
AKT1	✓			v1
AKT2	✓		✓	v1
AKT3	✓	✓		v1
ALK	✓	✓	✓	v1
ALOX12B	✓			v2
AMER1	✓			v2
APC	✓			v1
AR	✓	✓	✓	v1
ARAF	✓			v1
ARFRP1	✓			v2
ARID1A	✓			v1
ARID1B	✓			v2
ARID2	✓			v2
ASXL1	✓			v2
ATM	✓		✓	v1
ATR	✓			v1
ATRX	✓			v1
AURKA	✓			v2
AURKB	✓			v2
AXIN1	✓			v2
AXIN2	✓			v2
AXL	✓	✓		v1
B2M	✓			v1
BAP1	✓			v1
BARD1	✓			v1
BCL2		✓		v1
BCL2L1	✓			v2
BCL2L11	✓			v2
BCOR	✓			v2
BCORL1	✓			v2
BLM	✓			v1
BMPR1A	✓			v1
BRAF	✓	✓	✓	v1

continue...

...Continuation

Table 1S. Genes selected for variant reporting. Genes were initially reported using version 1 (v1, 159 genes) and, from June 2023 onwards, with version 2 (v2, 351 genes, including all genes in v1 plus additional clinically relevant targets)

Genes selected for variant reporting	DNA	Fusion	Amplification	Version
BRCA1	√	√	√	v1
BRCA2	√	√	√	v1
BRIP1	√			v1
C11ORF30 (EMSY)	√			v2
CASP8	√			v2
CBFB	√			v2
CCND1			√	v1
CCND3			√	v1
CCNE1			√	v1
CDC73	√			v1
CDH1	√			v1
CDK12	√			v1
CDK4	√	√	√	v1
CDK6			√	v1
CDKN1A	√			v2
CDKN1B	√			v1
CDKN2A	√			v1
CDKN2B	√			v1
CDKN2C	√			v2
CHD4	√			v2
CHEK1	√		√	v1
CHEK2	√		√	v1
CIC	√			v1
CREBBP	√			v2
CSF1R		√		v1
CSNK1A1	√			v2
CTCF	√			v2
CTLA4	√			v2
CTNNA1	√			v2
CTNNB1	√			v1
CUL3	√			v2
CXCR4	√			v2
CYLD	√			v2
DAXX	√			v2
DDR2	√			v2
DICER1	√			v2
EED	√			v2
EGFR	√	√	√	v1
EIF1AX	√			v2
EML4		√		v1
EP300	√			v2
EPCAM	√			v1
EPHA3	√			v2
EPHA7	√			v1
EPHB1	√			v2
ERBB2	√	√	√	v1
ERBB3	√		√	v1

continue...

...Continuation

Table 1S. Genes selected for variant reporting. Genes were initially reported using version 1 (v1, 159 genes) and, from June 2023 onwards, with version 2 (v2, 351 genes, including all genes in v1 plus additional clinically relevant targets)

Genes selected for variant reporting	DNA	Fusion	Amplification	Version
ERBB4	√			v2
ERCC1			√	v2
ERCC2	√		√	v1
ERCC3	√			v2
ERCC4	√			v2
ERCC5	√			v2
ERG		√		v1
ERRFI1	√			v2
ESR1	√	√	√	v1
ETS1		√		v1
ETV1		√		v1
ETV4		√		v1
ETV5		√		v1
EWSR1		√		v1
FAM175A (ABRAXAS1)	√			v2
FANCA	√			v1
FANCC	√			v2
FANCD2	√			v2
FANCE	√			v2
FANCF	√			v2
FANCG	√			v2
FANCI	√			v2
FANCL	√			v1
FAT1	√			v2
FBXW7	√			v2
FGF1			√	v2
FGF10			√	v2
FGF14			√	v2
FGF19			√	v2
FGF2			√	v2
FGF23	√		√	v2
FGF3			√	v2
FGF4			√	v1
FGF5			√	v2
FGF6			√	v2
FGF7			√	v2
FGF8			√	v2
FGF9			√	v2
FGFR1	√	√	√	v1
FGFR2	√	√	√	v1
FGFR3	√	√	√	v1
FGFR4	√	√	√	v1
FH	√			v1
FLCN	√			v1
FLI1		√		v1
FLT1		√		v1
FLT3		√		v1

continue...

...Continuation

Table 1S. Genes selected for variant reporting. Genes were initially reported using version 1 (v1, 159 genes) and, from June 2023 onwards, with version 2 (v2, 351 genes, including all genes in v1 plus additional clinically relevant targets)

Genes selected for variant reporting	DNA	Fusion	Amplification	Version
FOXA1	√			v2
FOXL2	√			v1
FUBP1	√			v1
FYN	√			v2
GABRA6	√			v2
GATA3	√			v2
GEN1	√			v2
GNA11	√			v1
GNAQ	√			v1
GNAS	√			v1
GPR124 (ADGRA2)	√			v2
GPS2	√			v2
GRIN2A	√			v2
GRM3	√			v2
H3F3A	√			v1
H3F3B	√			v2
H3F3C (H3-5)	√			v2
HIST1H3A (H3C1)	√			v2
HIST1H3B (H3C2)	√			v2
HIST1H3C (H3C3)	√			v2
HIST1H3G (H3C8)	√			v2
HIST1H3I (H3C11)	√			v2
HIST1H3J (H3C12)	√			v2
HIST2H3D (H3C13)	√			v2
HIST3H3 (H3-4)	√			v2
HNF1A	√			v2
HOXB13	√			v1
HRAS	√			v1
HSD3B1	√			v2
IDH1	√			v1
IDH2	√			v1
IFNGR1	√			v2
INHA	√			v2
INHBA	√			v2
INPP4A	√			v2
INPP4B	√			v2
IRS1	√			v2
IRS2	√			v2
JAK1	√			v2
JAK2	√	√	√	v1
JAK3	√			v2
JUN	√			v2
KDM5C	√			v2
KDM6A	√			v1
KDR	√	√		v1
KEAP1	√			v1
KIF5B		√		v1
KIT	√	√	√	v1

continue...

...Continuation

Table 1S. Genes selected for variant reporting. Genes were initially reported using version 1 (v1, 159 genes) and, from June 2023 onwards, with version 2 (v2, 351 genes, including all genes in v1 plus additional clinically relevant targets)

Genes selected for variant reporting	DNA	Fusion	Amplification	Version
KLF4	√			v2
KMT2B (MLL4)	√			v2
KMT2C (MLL3)	√			v2
KMT2D (MLL2)	√			v2
KRAS	√		√	v1
LAMP1			√	v2
LATS1	√			v2
LATS2	√			v2
LMO1	√			v2
LRP1B	√			v2
LYN	√			v2
LZTR1	√			v2
MAP2K1	√			v1
MAP2K2	√			v1
MAP2K4	√			v2
MAP3K1	√			v2
MAP3K13	√			v2
MAP3K4	√			v2
MAPK1	√			v2
MAPK3	√			v2
MAX	√			v1
MDC1	√			v2
MDM2	√		√	v1
MDM4			√	v2
MED12	√			v2
MEN1	√			v1
MET	√	√	√	v1
MGA	√			v2
MITF	√			v1
MLH1	√			v1
MLL		√		v1
MLL (KMT2A)	√			v2
MLLT3		√		v1
MRE11A	√			v1
MSH2	√	√		v1
MSH3	√			v2
MSH6	√			v1
MTOR	√			v1
MUTYH	√			v1
MYC		√	√	v1
MYCL			√	v2
MYCN			√	v2
MYOD1	√			v2
NBN	√			v1
NF1	√			v1
NF2	√			v1
NFE2L2	√			v1
NFKBIA	√			v2

continue...

...Continuation

Table 1S. Genes selected for variant reporting. Genes were initially reported using version 1 (v1, 159 genes) and, from June 2023 onwards, with version 2 (v2, 351 genes, including all genes in v1 plus additional clinically relevant targets)

Genes selected for variant reporting	DNA	Fusion	Amplification	Version
NKX2-1	√			v2
NOTCH1		√		v1
NOTCH2		√		v1
NOTCH3		√		v1
NRAS	√		√	v1
NRG1		√	√	v1
NSD1	√			v2
NTRK1	√	√		v1
NTRK2	√	√		v1
NTRK3	√	√		v1
PALB2	√			v1
PARK2 (PRKN)	√			v2
PARP1	√			v2
PAX3		√		v1
PAX7		√		v1
PBRM1	√			v1
PDGFRA	√	√	√	v1
PDGFRB	√	√	√	v1
PHOX2B	√			v1
PIK3C2B	√			v2
PIK3C2G	√			v2
PIK3C3	√			v2
PIK3CA	√	√	√	v1
PIK3CB	√		√	v2
PIK3CD	√			v2
PIK3CG	√			v2
PIK3R1	√			v1
PIK3R2	√			v2
PIK3R3	√			v2
PMS1 (MLH2)	√			v2
PMS2	√			v1
POLD1	√			v1
POLE	√			v1
PPARG	√	√		v1
PPP2R1A	√			v2
PPP2R2A	√			v2
PPP6C	√			v2
PREX2	√			v2
PRKAR1A	√			v2
PRKDC	√			v2
PTCH1	√			v1
PTEN	√		√	v1
PTPN11	√			v2
PTPRD	√			v2
PTPRS	√			v2
PTPRT	√			v2
RAB35	√			v2
RAC1	√			v1

continue...

...Continuation

Table 1S. Genes selected for variant reporting. Genes were initially reported using version 1 (v1, 159 genes) and, from June 2023 onwards, with version 2 (v2, 351 genes, including all genes in v1 plus additional clinically relevant targets)

Genes selected for variant reporting	DNA	Fusion	Amplification	Version
RAD21	√			v2
RAD50	√			v1
RAD51	√			v1
RAD51B	√			v1
RAD51C	√			v1
RAD51D	√			v1
RAD52	√			v2
RAD54L	√			v1
RAF1	√	√	√	v1
RARA	√			v2
RASA1	√			v2
RB1	√			v1
RBM10	√			v2
RECQL4	√			v2
RET	√	√	√	v1
RHEB	√			v2
RHOA	√			v2
RICTOR			√	v1
RIT1	√			v2
RNF43	√			v2
ROS1	√	√		v1
RPS6KB1		√	√	v1
RPS6KB2	√			v2
SDHA	√			v1
SDHAF2	√			v1
SDHB	√			v1
SDHC	√			v1
SDHD	√			v1
SETD2	√			v2
SF3B1	√			v2
SLX4	√			v2
SMAD2	√			v2
SMAD3	√			v2
SMAD4	√			v1
SMARCA4	√			v1
SMARCB1	√			v1
SMARCD1	√			v2
SMC1A	√			v2
SMC3	√			v2
SMO	√			v1
SOCS1	√			v2
SOX17	√			v2
SPEN	√			v2
SPOP	√			v2
SPTA1	√			v2
SRC	√			v2
STAG1	√			v2
STAG2	√			v2

continue...

...Continuation

Table 1S. Genes selected for variant reporting. Genes were initially reported using version 1 (v1, 159 genes) and, from June 2023 onwards, with version 2 (v2, 351 genes, including all genes in v1 plus additional clinically relevant targets)

Genes selected for variant reporting	DNA	Fusion	Amplification	Version
STAT3	√			v2
STK11	√			v1
SUFU	√			v1
SUZ12	√			v2
SYK	√			v2
TAF1	√			v2
TBX3	√			v2
TCEB1 (ELOC)	√			v2
TCF7L2	√			v2
TERT	√			v1
TFRC			√	v2
TGFBR1	√			v2
TGFBR2	√			v2
TMEM127	√			v1
TMPRSS2		√		v1
TOP1	√			v2
TP53	√			v1
TRAF2	√			v2
TRAF7	√			v2
TSC1	√			v1
TSC2	√			v1
TSHR	√			v2
U2AF1	√			v2
VHL	√			v1
WISP3 (CCN6)	√			v2
WT1	√			v1
XRCC2	√			v1
ZBTB20				v2
ZFHX3	√			v2

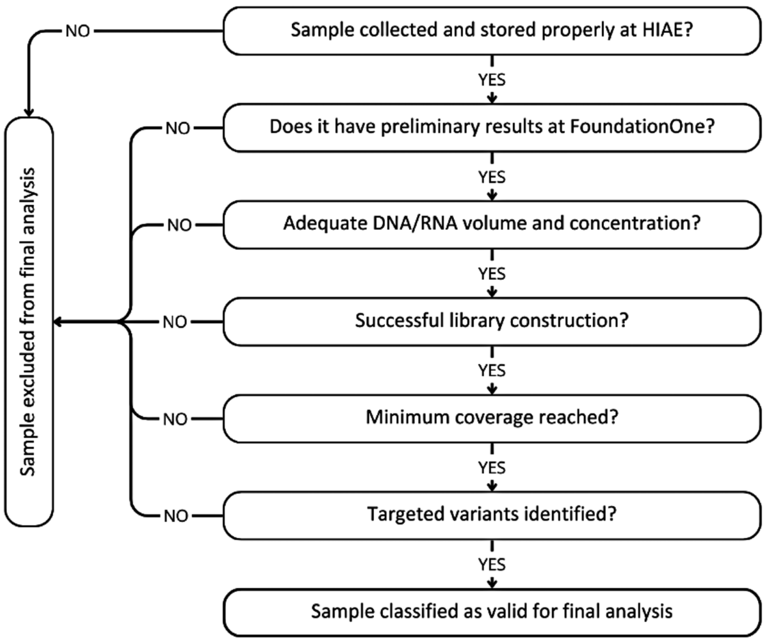

HIAE: Hospital Israelita Albert Einstein.

Figure 1S. Flowchart of sample eligibility for validation analysis

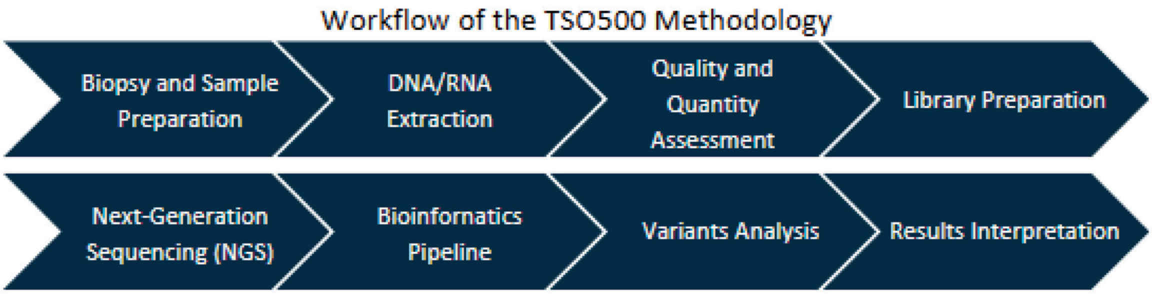

Figure 2S. Workflow of the TSO500 methodology

Table 2S. Sample types used for the TSO500 accuracy validation

Sample	DNA	RNA
Bone	1	-
Brain	5	8
Breast	2	1
Colon	2	-
Endometrium	1	-
Gastroesophageal junction	1	-
Liver	8	3
Lung	7	1
Lymph node	1	-
Mediastinum	1	-
Omentum	1	-
Ovary	2	-
Palato	1	-
Pancreas	2	1
Paravetebral muscle	-	1
Parotid gland	1	1
Pleura	1	-
Prostate	1	1
Skin	3	2
Soft tissue (arm)	1	-
Thyroid	2	1
Unknown	-	13
Uterus	2	1
Total samples	46	34

Table 3S. Characteristics of the 37 cases negative for oncogenic or likely oncogenic variants. The table includes tumor type, tumor mutational burden (mut/Mb), and microsatellite instability status

ID	Diagnosis	TMB (mut/Mb)	MSI status
47	Colorectal adenocarcinoma	5.5	MSS
87	Pancreatic adenocarcinoma	5.5	MSS
100	Pancreatic adenocarcinoma	3.14	MSS
134	Prostate adenocarcinoma	0	MSS
135	Prostate adenocarcinoma	1.57	MSS
201	Lung adenocarcinoma	0.78	MSS
208	Pancreatobiliary tract adenocarcinoma	7.04	MSS
216	Pancreatobiliary tract adenocarcinoma	0.8	MSS
228	Gastric adenocarcinoma	5.49	MSS
234	Adenocarcinoma NOS	4.69	MSS
244	Merkel cell carcinoma	2.35	MSS
261	Ovarian carcinoma	0.8	MSS
267	Ovarian carcinoma	0	MSS
269	Penile carcinoma	3.9	MSS
285	Anal canal squamous cell carcinoma	3.9	MSS
307	Renal carcinoma	3.93	MSS
312	Renal carcinoma	0	MSS
320	Carcinoma NOS	4.7	MSS
329	Carcinoma NOS	0.78	MSS
331	Carcinoma NOS	0.8	MSS
361	Cholangiocarcinoma	3.13	MSS
368	Ependymoma	1.57	MSS
374	Glioma	3.13	MSS
394	Glioma	4.69	MSS
403	Epithelioid hemangioendothelioma	0.78	MSS
418	NA	8.63	NA
420	NA	0	NA
423	Neuroblastoma	6.2	MSS
424	Neuroblastoma	1.56	MSS
425	Sarcoma	1.56	MSS
430	Sarcoma	2.4	MSS
435	Sarcoma	3.98	MSS
437	Sarcoma	0.78	MSS
438	Sarcoma	0.78	MSS
445	Sarcoma	NA	MSS
447	Sarcoma	4.01	MSS
453	Juxtaglomerular cell tumor	1.56	MSS

sMSS: microsatellite stable; NA: not applicable; MSI: microsatellite instability.

Table 4S. Characteristics of all samples with oncogenic/likely oncogenic variants

ID	Oncogenic/ Likely oncogenic variants	TMB (mut/Mb)	MSI status	Percentage of unstable sites	Diagnosis	Sample site
1	ERBB2 c.929 C >A p.(Ser310 Tyr), Amplification EGFR	0.78	Not detected	2.94	Ampullary adenocarcinoma	Liver
2	NRAS Q61K, TP53 R282W	6.28	Not detected	1.43	Colorectal adenocarcinoma	Abdominal wall
3	Fusion GOPC::ROS	0	Not detected	0	Colorectal adenocarcinoma	Lymph node
4	KRAS c.34 G>T p.(Gly12Cys), TP53 c.369_370delT T c.(Cys124 Hisfs24), SMAD4 c.1570 T>C p.(Trp524 A rg), APC c.3980 C >G (p.Ser1327 Ter)	9.49	Not detected	3.45	Colorectal adenocarcinoma	Lung
5	CIC c.3452dupC p.(Ser1154 GlnfsTer40), KRAS c.35 G>T p.(Gly12 Val), TP53 c.97-1 G>A, SMAD4 c.1081 C >A p.(A rg361 Ser), APC c.3520delG p.(A sp1174 Ilefs*8)	7.03	Not detected	3.57	Colorectal adenocarcinoma	Duodenum
6	KRAS c.38 G>A p.(Gly13 A sp) e c.64 C >A p.(Gln22 Lys), TP53 c.713 G>A p.(Cys238 Tyr)	9.37	Not detected	3.51	Colorectal adenocarcinoma	Peritoneum
7	NRAS c.181 C >A p.(Gln61 Lys), TP53 c.743 G>A p.(A rg248 Gln), SMAD4 c.804 G>A p.(Trp268 Ter)	10.95	Not detected	1.02	Colorectal adenocarcinoma	Rectum
8	BRAF c.1799 T>A (p.Val600 Glu), TP53 c.734 G>T (p.Gly245 Val), APC c.4660 G>T (p.Glu1554 Ter) e c.4033 G>T (p.Glu1345 Ter)	3.9	Not detected	6	Colorectal adenocarcinoma	Peritoneum
9	KRAS c.436 G>A p.(A la146 Thr), APC c.730-1 G>A, TP53 c.637 C >T p.(A rg213 Ter)	8.65	Not detected	0.88	Colorectal adenocarcinoma	Lung
10	TP53c.645T>G p.S215R	0	Not detected	2.33	Colorectal adenocarcinoma	Colon
11	APC c.2626 C >T p.(Arg876 Ter), c.3922 A >T p.(Lys1308 Ter); TP53 c.859 G>T p.(Glu287 Ter), c.731delG:p.(Gly244 A lafs*3)	4.19	Indeterminate	0	Colorectal adenocarcinoma	Rectosigmoid
12	KRAS c.35G>T p.(Gly12Val); APC c.3566delC p.(Ser1189TyrfsTer76), c.3927_3931del p.(Glu1309AspfsTer4); MLH1 c.15delA p.(Val7LeufsTer10); TP53 c.632C>T p.(Thr211Ile); MUTYH c.1145G>A p.(Gly382Asp); KDM6A c.279_334del p.(Asp94IlefsTer13); EPHA7 c.2847C>A p.(Tyr949Ter)	6.36	Not detected	4.17	Colorectal adenocarcinoma	Liver
13	KRAS c.35 G>T p.(Gly12 Val); TP53 c.919+1 G>A; SMAD4 c.1156 G>T p.(Gly386 Cys), c.1487 G>A p.(A rg496 His)	7.04	Not detected	3.42	Colorectal adenocarcinoma	Colon
14	PIK3CA c.3140 A >T p.(His1047 Leu); TP53 c.743 G>A p.(A rg248 Gln); APC c.4192_4193delA G p.(A rg1399 PhefsTer9)	5.4	Not detected	2.33	Colorectal adenocarcinoma	Rectosigmoid
15	KRAS c.35 G>T p.(Gly12 Val); TP53 c.524 G>A p.(A rg175 His)	4	Not detected	4.49	Colorectal adenocarcinoma	Colon
16	KRAS c.38 G>A p.(Gly13 Asp); ERBB2 c.929 C >T p.(Ser310 Phe); TP53 c.733 G>A p.(Gly245 Ser); APC c.4463dupT p.(Leu1488 PhefsTer26); MYC Amplification	6.2	Not detected	5.15	Colorectal adenocarcinoma	Rectosigmoid
17	BRAF V600E; BRCA1 I986fs; PTEN A192fs; TP53 R156H, R175H	6.3	Not detected	0.85	Colorectal adenocarcinoma	Liver
18	KRAS G13D; APC R1114*, F1491fs	6.3	Not detected	1.83	Colorectal adenocarcinoma	Colon
19	BRCA2 K944*; KRAS G12V; APC S1415fs; TP53 R282W	13.3	Not detected	3.33	Colorectal adenocarcinoma	Pelvis
20	KRAS G13D; APC c.834+1G>A; TP53 R273H; SMAD4 G365D	5.5	Not detected	2.56	Colorectal adenocarcinoma	Rectosigmoid
21	BRAF V600E; BLM N515fs; NBN R466fs; TP53 R213fs; RAD50 K722fs; FLCN H429fs; GNAS R201C; FOXL2 G269fs; Amplification RICTOR; Amplification NRG1	85.6	Detected	74.26	Colorectal adenocarcinoma	Brain
22	KRAS Q61L; TP53 R248Q; APC H1490fs	2.3	Not detected	3.28	Colorectal adenocarcinoma	Cecum
23	APC Y1078*, Q1367*; PIK3R1 H450_E451delinsQ	6.3	Not detected	1.33	Colorectal adenocarcinoma	Rectosigmoid

continue...

...Continuation

Table 4S. Characteristics of all samples with oncogenic/likely oncogenic variants

ID	Oncogenic/ Likely oncogenic variants	TMB (mut/Mb)	MSI status	Percentage of unstable sites	Diagnosis	Sample site
24	PIK3CA E542K; KRAS G12V; APC R332*, R805*, D1519fs; MSH6 F1088fs; TP53 Y220C; Amplification RICTOR	5.5	Not detected	25	Colorectal adenocarcinoma	Colon
25	APC S837*; TP53 A70fs	6.3	Not detected	2.41	Colorectal adenocarcinoma	Brain
26	APC E1306*, TP53 R248Q, SMAD4 R496H	7.03	Not detected	3.39	Colorectal adenocarcinoma	Liver
27	PIK3CA G118D, KRAS G12S, APC Q1406*, TP53 R282W	6.3	Not detected	6.36	Colorectal adenocarcinoma	Colon
28	KRAS G12D, TP53 A161D, APC S1344*	10.17	Not detected	2.88	Colorectal adenocarcinoma	Liver
29	KRAS G12V, TP53 R273H, APC E1295*	6.27	Not detected	1.15	Colorectal adenocarcinoma	Liver
30	PIK3CA E545K, KRAS A59E, APC F1491fs, TP53 R273H	8.6	Not detected	5.22	Colorectal adenocarcinoma	Liver
31	NRAS Q61R, TP53 M246I	0.8	Indeterminate	0	Colorectal adenocarcinoma	Colon
32	APC R876*, S1501fs; ERBB3 A232V; TP53 A159fs; Amplification NRAS	8.6	Not detected	1.02	Colorectal adenocarcinoma	Liver
33	APC R876*, ERBB3 A232V, TP53 A159fs, Amplification NRAS, Amplification MYC	0	Not detected	1.03	Colorectal adenocarcinoma	Lung
34	APC R232*, CDKN1B D51fs, KRAS G12D, TP53 G266E	4.7	Not detected	1.89	Colorectal adenocarcinoma	Colon
35	KRAS G12V; APC Y1135*, Q1477*; SMAD4 W524C; TP53 R273H	4.7	Not detected	5.26	Colorectal adenocarcinoma	Liver
36	KRAS G13D; SMAD4 L536R; APC R1314Sfs*7; TP53 L145Q, R248W	5.53	Not detected	2.17	Colorectal adenocarcinoma	Rectosigmoid
37	BRAF V600E; CHEK2 A540fs; ATM L312fs; CDH1 S70fs, P126fs, B2M L15fs; BLM N515fs; NBN R466fs	66.5	Detected	54.55	Colorectal adenocarcinoma	Colon
38	KRAS A146T, SMAD4 L540fs	7	Not detected	1.8	Colorectal adenocarcinoma	Colon
39	KRAS G12D, TP53 R175H	7.9	Not detected	0	Colorectal adenocarcinoma	Rectosigmoid
40	APC M717*, TP53 E258K	5.58	Not detected	0	Colorectal adenocarcinoma	Rectosigmoid
41	PIK3CA H1047R, ARID1A Q1454fs	2.36	Not detected	0	Colorectal adenocarcinoma	Colon
42	BRAF V600E, TP53 R342*, SMAD4 D537V	4.7	Not detected	0	Colorectal adenocarcinoma	Colon
43	TP53 R273H	5.94	Not detected	0	Colorectal adenocarcinoma	Ovary
44	KRAS A146T, TP53 c.673- 50_757del	7.86	Not detected	0	Colorectal adenocarcinoma	Colon
45	KRAS G13D, APC L1489fs, TP53 C124fs, FBXW7 R505C	5.5	Not detected	0	Colorectal adenocarcinoma	Rectosigmoid
46	KRAS G12D, PTEN R130Q, PIK3R1 L570fs	7.06	Not detected	0	Colorectal adenocarcinoma	Liver
48	KRAS G12D, APC R876*, APC E1379*, TP53 C141W, TP53 Y163N	10.98	Not detected	0	Colorectal adenocarcinoma	Liver
49	APC G1288*, TP53 Q192*	5.5	Not detected	0	Colorectal adenocarcinoma	Liver
50	KRAS G12V, APC R564* , APC P1424fs, TP53 V216G	14.89	Not detected	0	Colorectal adenocarcinoma	Prostate
51	ERBB2 R678Q, NRAS G12V, SMAD4 P356del, APC S1411fs	1.6	Not detected	0	Colorectal adenocarcinoma	Rectum
52	KRAS G12D, APC Q663*, MAP3K1 c.483- 1G>A	3.13	Not detected	0	Colorectal adenocarcinoma	Liver
53	Amplification FGFR1, TP53 R248W	4.7	Not detected	4.11	Esophagogastric junction adenocarcinoma	Esophagogastric junction
54	Amplification KRAS, Amplification CDK4, Amplification MDM2	3.13	Not detected	3.75	Esophagogastric junction adenocarcinoma	Esophagogastric junction
55	APC Q1367*, KRAS G12V, VHL E52*, TP53 R283del, Amplification FGF9, Amplification FGF14, Amplification MYC (suspicious)	8.61	Not detected	0	Colon adenocarcinoma	Colon
56	APC S1180fs, T1301fs; TP53 V173L	3.9	Not detected	0.88	Colon adenocarcinoma	Lung

continue...

...Continuation

Table 4S. Characteristics of all samples with oncogenic/likely oncogenic variants

ID	Oncogenic/ Likely oncogenic variants	TMB (mut/Mb)	MSI status	Percentage of unstable sites	Diagnosis	Sample site
57	KRAS G12V, APC L1488fs, TP53 L257R, CDKN2B D86N, TERT promoter -124C>T	5.47	Not detected	3.45	Colon adenocarcinoma	Liver
58	KRAS G12D, TERT promoter -124C>T	12.54	Not detected	0	Colon adenocarcinoma	Right colon
59	APC R1450*, TP53R248W	7.82	Not detected	0	Colon adenocarcinoma	Colon
60	KRAS c.35G>A p.(Gly12Asp) subclonal	2.35	Not detected	4.81	Duodenal adenocarcinoma	Peritoneum
61	KRAS c.35 G>A p.(Gly12 A sp); TP53 c.103_119delinsA A G G p.(Leu35 Lysfs*5)	0	Not detected	1.98	Duodenal adenocarcinoma	Duodenum
62	KRAS Q61K, TP53 H179Y	1.56	Not detected	2.56	Duodenal adenocarcinoma	Duodenum
63	Amplification MET, TP53 R196*	6.3	Not detected	6.96	Esophageal adenocarcinoma	Esophagus
64	TP53 Y234S, Amplification CDK6	3.14	Not detected	0	Esophageal adenocarcinoma	Esophagus
65	NF1 L1304*, TP53 A70fs	2.35	Not detected	2.94	Ovary adenocarcinoma	Uterus
66	BRCA2 S1982Rfs*22, MSH6 R772W, TP53 R175H	2.3	Not detected	1.56	Ovary adenocarcinoma	Ovary
67	TP53 Q165*	6.3	Not detected	0	Ovary adenocarcinoma	Soft tissue
68	CDKN2A Y44X, KRAS Q61H, TP53 A88Pfs	3.9	Not detected	0.87	Pancreatic adenocarcinoma	Pancreas
69	TP53 R282W, KRAS G12V	5	Not detected	1.39	Pancreatic adenocarcinoma	Pancreas
70	KRAS G12R, ATM Q414fs	14.22	Not detected	1.71	Pancreatic adenocarcinoma	Peritoneum
71	KRA S c.35 G>A p.(Gly12Asp), TP53 c.1146delA p.(Lys382 A snfs*40), ARID1A c.2296delC .p.(Gln766 Serfs*67), KEAP1 c.1153 G>A p.(A sp385 Asn), MLH1 c.1151 T>A p.(Val384 Asp)	26.9	Detected	29.82	Pancreatic adenocarcinoma	Duodenum
72	TP53 c.810delT p.(Phe270 Leufs*75), KRAS c.35 G>A p.(Gly12 A sp), SMAD4 c.1082_1096del p.(A rg361_Gly365del), ARID1A c.2179_2189del p.(A rg727 Trpfs*86), MEN1 c.669+3 A >G, Amplification CDK4	7.14	Not detected	2.5	Pancreatic adenocarcinoma	Pancreas
73	KRAS c.35 G>A p.(Gly12 Asp), TP53 c.488 A >G p.(Tyr163 Cys)	9.46	Not detected	0	Pancreatic adenocarcinoma	Lymph node
74	KRAS c.35 G>A p.(Gly12 A sp), ARID1A c.2143dupT p.(Ser715 Phefs*102), TP53 c.337 T>G p.(Phe113 Val)	11.21	Indeterminate	2.63	Pancreatic adenocarcinoma	Pancreas
75	KRAS c.35 G>A p.(Gly12 Asp)	0	Not detected	2.15	Pancreatic adenocarcinoma	Pancreas
76	KRAS c.35 G>T p.(Gly12 Val)	3.92	Not detected	2.33	Pancreatic adenocarcinoma	Ovary
77	KRAS G12D, TP53 R249S, FH K477dup	1.57	Not detected	0	Pancreatic adenocarcinoma	Liver
78	KRAS G12D, TP53 V173M, SMAD4 W268*, Fusion RPS6KB1-VMP1	2.35	Not detected	2.97	Pancreatic adenocarcinoma	Liver
79	KRAS c.35 G>T p.(Gly12 Val); TP53 c.524 G>A p.(A rg175 His)	6.28	Not detected	0	Pancreatic adenocarcinoma	Pancreas
80	KRAS G12R; TP53 Y234C	0.8	Not detected	1.92	Pancreatic adenocarcinoma	Liver
81	KRAS G12C; TP53 Y103*	3.9	Not detected	0.81	Pancreatic adenocarcinoma	Pancreas
82	CDKN2A R80X; KRAS G12D; TP53 L330Hfs*7	3.9	Not detected	3.3	Pancreatic adenocarcinoma	Liver
83	CDKN2A R87fs; KRAS G12V; TP53 Y163C; ATR K1665fs	5.5	Not detected	1.8	Pancreatic adenocarcinoma	Lung
84	CDKN2A N71fs; KRAS G12D; TP53 V173L	3.1	Not detected	3.77	Pancreatic adenocarcinoma	Bone
85	KRAS Q61H; TP53 Y126N; VHL P81S	3.9	Not detected	0.83	Pancreatic adenocarcinoma	Liver
86	TP53 L257R; SMAD4 P198Qfs*4; KRAS G12V	4.72	Not detected	1.33	Pancreatic adenocarcinoma	Pancreas
88	KRAS G12D (subclonal)	0	Not detected	2.46	Pancreatic adenocarcinoma	Pancreas
89	KRAS G12D, SMAD4 R380K, TP53 P153fs	4.69	Not detected	4.17	Pancreatic adenocarcinoma	Pancreas
90	KRAS G12R, SMAD4 R515T,TP53 E258K	4.69	Not detected	3.33	Pancreatic adenocarcinoma	Liver
91	CDKN2A W15fs, KRAS G12D, TP53 R337H	1.56	Not detected	0	Pancreatic adenocarcinoma	Lung
92	PTCH1 E2fs, ATM c.185+1G>C, ARID1A P695fs, KRAS G12D	3.13	Not detected	2.56	Pancreatic adenocarcinoma	Peritoneum

continue...

...Continuation

Table 4S. Characteristics of all samples with oncogenic/likely oncogenic variants

ID	Oncogenic/ Likely oncogenic variants	TMB (mut/Mb)	MSI status	Percentage of unstable sites	Diagnosis	Sample site
93	CDKN2A H83Y, KRAS G12D, TP53 F109V, Amplification MYC	1.57	Not detected	0.92	Pancreatic adenocarcinoma	Liver
94	KRAS G12D, TP53 N247I, FANCA c.4011- 1G>C	3.15	Not detected	4.82	Pancreatic adenocarcinoma	Pancreas
95	KRAS G12R, TP53 K321Nfs	1.56	Not detected	5.83	Pancreatic adenocarcinoma	Pancreas
96	KRAS G12V, TP53 P128del, Amplification KRAS, Amplification CDK6	2.52	Not detected	2.38	Pancreatic adenocarcinoma	Liver
97	KRAS G12D, TP53 N288fs, CDKN2A P81S	4.7	Not detected	1.79	Pancreatic adenocarcinoma	Liver
98	KRAS G12R, TP53 P278T	0.78	Not detected	2.47	Pancreatic adenocarcinoma	Retroperitoneum
99	KRAS G12L, SMAD4 Q180*	7.07	Not detected	4.76	Pancreatic adenocarcinoma	Pancreas
101	KRAS G12R, TP53 E258*	6.31	Not detected	0	Pancreatic adenocarcinoma	Pancreas
102	KRAS G12D, GNAS R201H	3.13	Not detected	0	Pancreatic adenocarcinoma	Lymph node
103	KRAS G12V, TP53 E298fs	2.36	Not detected	0	Pancreatic adenocarcinoma	Pancreas
104	KRAS Q61R, SMAD4 P293fs	5.5	Not detected	0	Pancreatic adenocarcinoma	Omentum
105	KRAS G12D, TP53 R342*	3.41	Indeterminate	0	Pancreatic adenocarcinoma	Liver
106	KRAS G12V, SMAD4 K507N, TP53 R283fs	3.14	Not detected	0	Pancreatic adenocarcinoma	Pancreas
107	KRAS G12R	0.79	Not detected	0	Pancreatic adenocarcinoma	Pancreas
108	KRAS G12D, TP53 R282W, Amplification CDK6 (suspicious), Amplification MET (suspicious)	0	Not detected	0	Pancreatic adenocarcinoma	Liver
109	KRAS G12D, TP53 R175H, TGFBR2 S150*, TGFBR2F279fs	4.03	Not detected	0	Pancreatic adenocarcinoma	Pancreas
110	CDKN2A R58*, KRAS G12V, TP53 P278L, SMARCA4 T910M, TGFBR2 R495fs	4.71	Not detected	0	Pancreatic adenocarcinoma	Pancreas
111	TERT c.-124C>T	3.15	Not detected	0	Parotid adenocarcinoma	Head and neck
112	AR c.2632 A >G p.(Thr878 Ala), Amplification CCND1, Amplification FGFR3	2.35	Not detected	1.1	Prostate adenocarcinoma	Lymph node
113	Fusion SLC45A3-ERG	5.5	Not detected	3.26	Prostate adenocarcinoma	Lymph node
114	TMPRSS2-ERG Fusion; BAP1 S623_K630del; AKT1 L52R; PIK3CA E545K; SMAD4 R515S	7.1	Not detected	0	Prostate adenocarcinoma	Prostate
115	ATM c.67 C >T p.(Arg23 Ter) e c.8287 C >T p.(Arg2763 Ter); Fusion ERG (NM_001243432)-TMPRSS2 (NM_005656)	5.5	Not detected	3.03	Prostate adenocarcinoma	Prostate
116	TP53 R273C ; Fusion ETV1-SLC30A4	2.4	Not detected	3.03	Prostate adenocarcinoma	Prostate
117	TP53 c.844 C >T p.(Arg282 Trp); AR Amplification; MYC Amplification	0	Not detected	1.71	Prostate adenocarcinoma	Lymph node
118	Fusion SND1-BRAF; TP53 C135W	7.03	Not detected	0.84	Prostate adenocarcinoma	Liver
119	TP53 V73fs; Amplification CCND1; Fusion DDX5-ETV4	7.1	Not detected	2.17	Prostate adenocarcinoma	Prostate
120	Fusion TMPRSS2-ETV4	3.1	Not detected	0.85	Prostate adenocarcinoma	Prostate
121	BRCA2 c.7436-2A>C; HRAS Q61R; TP53 R280S; Amplification AR	7.8	Not detected	4.17	Prostate adenocarcinoma	Lymph node
122	BRIP1 P47A, TP53 L344P, Fusion TMPRSS2- ERG	1.56	Not detected	1.11	Prostate adenocarcinoma	Prostate
123	TP53 L194R; APC T1459fs	3.13	Not detected	0.9	Prostate adenocarcinoma	Prostate
124	TP53 L25fs, Amplification CCNE1	3.14	Not detected	3.26	Prostate adenocarcinoma	Lymph node
125	Fusion TMPRSS2-ERG, TP53 c.919+2dup, Amplification CCNE1	5.5	Not detected	5.56	Prostate adenocarcinoma	Lymph node
126	ATM c.7630- 2A>C, Fusion TMPRSS2- ERG, APC Q1627Kfs*, TP53 I255F	2.36	Not detected	1.82	Prostate adenocarcinoma	Brain
127	BRCA2 E1299X;CTNNB1 D32N	7.8	Not detected	1.79	Prostate adenocarcinoma	Prostate
128	BRCA2 K2777fs	4.69	Not detected	0.89	Prostate adenocarcinoma	Prostate
129	ERCC2 F538_A550del, CTNNB1 T41A, Amplification PIK3CA, RB1 K63fs	Indeterminate	Not detected	7.41	Prostate adenocarcinoma	Lung

continue...

...Continuation

Table 4S. Characteristics of all samples with oncogenic/likely oncogenic variants

ID	Oncogenic/ Likely oncogenic variants	TMB (mut/Mb)	MSI status	Percentage of unstable sites	Diagnosis	Sample site
130	PMS2 K301N	4.7	Not detected	0	Prostate adenocarcinoma	Prostate
131	CHEK2 S428F, PIK3CA E726K, Amplification FGFR1, TP53 H214R, Amplification MYC	2.34	Not detected	6.67	Prostate adenocarcinoma	Prostate
132	PTEN R15fs	3.13	Not detected	0.93	Prostate adenocarcinoma	Lung
133	PALB2 L32fs, RB1 K289*	7.86	Not detected	2.44	Prostate adenocarcinoma	Prostate
136	CHEK2 c.320- 2A>G, Fusion RP11- 35609.1::ETV1	0	Not detected	0	Prostate adenocarcinoma	Prostate
137	AKT1 E17K	0	Not detected	0	Prostate adenocarcinoma	Prostate
138	ATM Y1470* , KDM6A c.3285- 2A>G, AR T878A, AR L702H, AR F877L, RNF43 G659fs, STAT3 R382W, KMT2C F4496fs, MSH3 K383Rfs*32, MSH3 K383Gfs*20, ALOX12B V527M, FUBP1 S11Lfs, KMT2D A2119fs, KMT2D P2354fs, CREBBP V1998*, APC K1878fs, AKT1 Q79	69.84	Detected	0	Prostate adenocarcinoma	Lymph node
139	KRAS c.34 G>T p.(Gly12 Cys), STK11 c.241 A >T p.(Lys81 Ter), KEAP1 c.958 C >T p.(A rg320 Trp), SMARCA4 c.1419+1 G>A	3.94	Not detected	0	Lung adenocarcinoma	Lung
140	KRAS c.35 G>T p.Gly12 Val; STK11 c.402delT p.(Cys134 Trpfs*27); ATRX c.2892dupT p.(A la965 Cysfs*12)	Indeterminate	Not detected	1.09	Lung adenocarcinoma	Lung
141	KRAS c.35 G>A p.(Gly12 Asp); CDKN2A c.151-1 G>T; TP53 c.535 C >T p.(His179 Tyr); PMS2 c.7 C >T p.(Arg3 Ter)	19.27	Not detected	1.61	Lung adenocarcinoma	Pelvis
142	KRAS c.34 G>T p.(Gly12 Cys); TP53 c.1010 G>A p.(A rg337 His); STK11 c.527 A >T p.(A sp176 Val)	7.85	Not detected	0.83	Lung adenocarcinoma	Pleura
143	KRAS c.35 G>A p.(Gly12 A sp); RAF1 c.786 T>A p.(Asn262 Lys)	9.4	Not detected	0	Lung adenocarcinoma	Lung
144	KRAS G12D; STK11 P281fs	10.9	Not detected	1.9	Lung adenocarcinoma	Liver
145	MET D1010N, exon skipping 14; TP53 R248W	16.5	Not detected	3.08	Lung adenocarcinoma	Colon
146	ERBB2 Y772_A775dup; CHEK2 S428F	2.3	Not detected	3.64	Lung adenocarcinoma	Lymph node
147	IDH2 R172S	3.1	Not detected	0	Lung adenocarcinoma	Liver
148	EGFR E746_A750del; PIK3CA H1047R	2.3	Not detected	3.33	Lung adenocarcinoma	Lung
149	Fusion KIF5B- RET; TP53 R248Q; KDM6A W1021*; Amplification MYC	5.5	Not detected	2.83	Lung adenocarcinoma	Lung
150	KEAP1 V155F; STK11 Y166fs	10.9	Not detected	1.74	Lung adenocarcinoma	Lung
151	ERBB2 C334F; PTCH1 c.655-1G>T; KRAS G13D; KEAP1 E218*; STK11 E165*; APC E1538*	7.8	Not detected	0.9	Lung adenocarcinoma	Lung
152	Fusion CD74-ROS1	0.8	Not detected	0.88	Lung adenocarcinoma	Lung
153	TP53 C242S, KEAP1 R272L	9.39	Not detected	1.82	Lung adenocarcinoma	Lung
154	Fusion CD74-NRG1	5.5	Not detected	3.25	Lung adenocarcinoma	Lung
155	Fusion ALK- EML4, TP53 E343*	1.59	Not detected	0	Lung adenocarcinoma	Peritoneum
156	CTNNB1 S45Y, TP53 Y126*, Fusion SDC4::ROS1	3.1	Not detected	0.89	Lung adenocarcinoma	Lung
157	MET exon 14 skipping, NF1 Y2285*	2.3	Not detected	1.27	Lung adenocarcinoma	Lung
158	CDKN2A L64_N71del, KDM6A S1061*, TP53 R249S	60.17	Not detected	0	Lung adenocarcinoma	Lung
159	PIK3CA H1047R, ATM c.8268+1G>A, Fusion TOMM20-AKT3	9.39	Not detected	1.05	Lung adenocarcinoma	Liver
160	NF1 c.6862 C >T p.(Gln2288 Ter); TERT c.-124 C >T; EPHB4-MET Fusion	12.51	Not detected	0.87	Lung adenocarcinoma	Lung
161	KRAS G12D (subclonal), PALB2 V989*	0	Not detected	2.54	Lung adenocarcinoma	Lung
162	EGFR A767_V769dup	0.78	Not detected	3.19	Lung adenocarcinoma	Lung
163	BRCA1 c.135- 2A>G, FANCL T367Nfs*, KRAS G12D, TP53 A159V, Amplification CCND1	5.48	Not detected	3.54	Lung adenocarcinoma	Pleura

continue...

...Continuation
Table 4S. Characteristics of all samples with oncogenic/likely oncogenic variants

ID	Oncogenic/ Likely oncogenic variants	TMB (mut/Mb)	MSI status	Percentage of unstable sites	Diagnosis	Sample site
164	MET exon 14 skipping	5.48	Not detected	1.77	Lung adenocarcinoma	Lung
165	KRAS G12V, TP53 R175H	5.47	Not detected	4.82	Lung adenocarcinoma	Lung
166	MSH2 G71R, TP53 R337H, Amplification RICTOR	3.13	Not detected	1.96	Lung adenocarcinoma	Liver
167	EGFR E746_A750del, TP53 c.994-1G>C	2.35	Not detected	0	Lung adenocarcinoma	Lung
168	EGFR E746_A750del, TP53 C176F	0	Not detected	1.98	Lung adenocarcinoma	Lung
169	KRAS G12C, STK11 E223*, TP53 R175C	7.04	Not detected	0.92	Lung adenocarcinoma	Lung
170	NF1 Q236*, STK11 C132fs, SMARCA4 Q987*, TP53 R156P	22.8	Not detected	2.6	Lung adenocarcinoma	Lymph node
171	EGFR A746_E750del, CTNNB1 S45F, Amplification MDM2	3.15	Not detected	0	Lung adenocarcinoma	Lymph node
172	EGFR L747_T751del, CHEK2 R346C	6.28	Not detected	6.28	Lung adenocarcinoma	Lung
173	EGFR L858R	8.6	Not detected	0	Lung adenocarcinoma	Lung
174	EGFR E746_A750del, TP53 L114*	2.4	Not detected	1.41	Lung adenocarcinoma	Liver
175	EGFR H773_V774dup	4.7	Not detected	0	Lung adenocarcinoma	Lung
176	EGFR L858R, Amplification EGFR, PIK3CA E542K, MUTYH G382D, TP53 L194F	7.08	Not detected	0	Lung adenocarcinoma	Lung
177	EGFR E746_A750del	1.59	Not detected	1.54	Lung adenocarcinoma	Lung
178	TP53 V272L	4.71	Not detected	3.33	Lung adenocarcinoma	Lung
179	CDKN1B E53Kfs	4.7	Not detected	2.33	Lung adenocarcinoma	Lung
180	ERBB2 Y772_A775dup, ARID1A D641Ifs*, TP53 R342P	3.16	Not detected	0	Lung adenocarcinoma	Pleura
181	MAP2K1 E102_I103del, TP53 H193L, Amplification AR	10.23	Not detected	0	Lung adenocarcinoma	Pleura
182	EGFR E746_A750del, Amplification EGFR (suspicious)	1.57	Not detected	0	Lung adenocarcinoma	Lung
183	EGFR E746_S752delinsV	2.36	Not detected	0	Lung adenocarcinoma	Lung
184	KRAS G12V, MAX D65fs	5.5	Not detected	0	Lung adenocarcinoma	Lung
185	TP53 A159V, PBRM1 Q235*	38.5	Not detected	0	Lung adenocarcinoma	Lymph node
186	EGFR L858R, Amplification EGFR (suspicious), TP53 Y236C	4.71	Not detected	0	Lung adenocarcinoma	Lung
187	KRAS G12C, PIK3CA E545K, CDKN1B Q65*	16.53	Not detected	0	Lung adenocarcinoma	Pleura
188	EGFR L858R, KRAS G12V, ARID1A Q586*, MSH2 G674A, TP53 M246V	7.85	Not detected	0	Lung adenocarcinoma	Lung
189	BRAF K601E, TP53 P72fs*	13.36	Not detected	0	Lung adenocarcinoma	Lung
190	EGFR L62R, EGFR G719A, Amplification EGFR, TP53 T125R, Amplification RICTOR, Amplification CCND3	12.54	Not detected	0	Lung adenocarcinoma	Lung
191	EGFR E746_A750del, TP53 I162N, Amplification EGFR (suspicious), Amplification MYC, Amplification CCND1, Amplification FGF3, Amplification FGF4	4.69	Not detected	0	Lung adenocarcinoma	Lung
192	EGFR L747_P753delinsS , MUTYH c.850-2A>G, BAP1 L65Wfs*7, BAP1 c.1983+1_1983+17del, ATM K1387Sfs*11, BCOR S620Vfs*51, Amplification MYC (suspicious)	11.75	Not detected	0	Lung adenocarcinoma	Omentum
193	KRAS G12A, TP53 V274L, Amplification MYC, Amplification CCND1, Amplification MYCN, Amplification FGF8, Amplification FGFR3, Amplification RET	9.39	Not detected	0	Lung adenocarcinoma	Lung
194	KRAS G12A	2.34	Not detected	0	Lung adenocarcinoma	Lung

continue...

...Continuation

Table 4S. Characteristics of all samples with oncogenic/likely oncogenic variants

ID	Oncogenic/ Likely oncogenic variants	TMB (mut/Mb)	MSI status	Percentage of unstable sites	Diagnosis	Sample site
195	KRAS G13E, STK11 D176N,CHEK2 G306A, SMARCA4 S1167*, KDM6A c.444-1G>C, RBM10 c.2362-2A>G	11.74	Not detected	0	Lung adenocarcinoma	Lymph node
196	CDKN2A H83Y, Amplification MDM2	0	Not detected	0	Lung adenocarcinoma	Lymph node
197	TP53 R175H, Amplification KRAS	3.13	Not detected	0	Lung adenocarcinoma	Lung
198	KRAS, STK11 Y131* , SMARCA4Q164Afs*10, EPHA3 c.2075- 2A>C	10.17	Not detected	0	Lung adenocarcinoma	Lung
199	EGFR L858R, TP53 S149fs	3.16	Not detected	0	Lung adenocarcinoma	Lung
200	EGFR L858R, TSC2 P1305fs, Amplification MDM2 (suspicious), Amplification CDK4 (suspicious)	5.5	Not detected	0	Lung adenocarcinoma	Lung
202	KRAS c.38 G>A p.(Gly13 A sp); ARID1A c.5707_5708delinsT p.(Pro1903 Ter); AKT1 c.49 G>A p.(Glu17 Lys); GNAS c.601 C >T p.(A rg201 Cys)	7.06	Not detected	1.85	Gallbladder adenocarcinoma	Gallbladder
203	ARID1A Q546*, KRAS G12A, TP53 G266E, APC I1307K	10.97	Not detected	2.5	Gallbladder adenocarcinoma	Gallbladder
204	BAP1 L682fs, STK11 c.290+1_290+10del, MEN1 W441R	4.86	Not detected	0	Gallbladder adenocarcinoma	Gallbladder
205	TP53 R282Q, RAC1 P29S	6.28	Not detected	0	Gallbladder adenocarcinoma	Gallbladder
206	KRAS G12D, TP53 R282W	1.57	Not detected	0	Pancreatobiliary adenocarcinoma	Liver
207	ERBB2 R678Q, Amplification; TP53 R248Q	10.23	Not detected	0	Pancreatobiliary adenocarcinoma	Bile ducts
209	KRAS c.35G>T p.(Gly12Val), TP53 c.743G>A p.(Arg248 Gln), SMAD4 c.1485dupT p.(Arg496 Serfs*31), STK11 c.274delG p.(Glu92 Arg fs*4)	2.35	Not detected	4.12	Pancreatobiliary adenocarcinoma	Liver
210	KRAS c.34G>C p.(Gly12Arg), ATM c.6807+1G>C	3.18	Not detected	0	Pancreatobiliary adenocarcinoma	Peritoneum
211	ATM c.5644 C >T p.(Arg1882 Ter), c.8666 A >C p.(Asp2889 Ala); KRAS c.35 G>A p.(Gly12 Asp)	3.14	Not detected	1.96	Pancreatobiliary adenocarcinoma	Liver
212	KRAS c.35 G>A p.(Gly12 A sp); TP53 c.524 G>A p.(Arg175 His)	1.56	Not detected	0.88	Pancreatobiliary adenocarcinoma	Liver
213	KRAS c.35 G>T p.(Gly12 Val); ATM c.9139 C >T p.(Arg3047 Ter)	0.78	Not detected	3.96	Pancreatobiliary adenocarcinoma	Peritoneum
214	CHEK2 c.470 T>C p.(Ile157 Thr); KRAS c.35 G>T p.(Gly12 Val); PTEN c.78_79+7del; TP53 c.524 G>A (p.Arg175 His)	1.57	Not detected	0	Pancreatobiliary adenocarcinoma	Liver
215	CDKN2A c.247 C >T p.(His83 Tyr); KRAS c.35 G>A p.(Gly12 A sp); TP53 c.112 C >T p.(Gln38 Ter); MUTYH c.1145 G>A p.(Gly382 Asp)	6.26	Not detected	1.69	Pancreatobiliary adenocarcinoma	Peritoneum
217	KRAS G12D; TP53 V73fs	4.7	Not detected	1.72	Pancreatobiliary adenocarcinoma	Liver
218	KRAS G12V; TP53 Y126H; RAD51 c.645- 12_645-1del; ERBB3 T355N	5.5	Not detected	4.24	Pancreatobiliary adenocarcinoma	Lung
219	KRAS G12D, CDKN2A G45fs, TP53 M237I	3.16	Not detected	2.56	Pancreatobiliary adenocarcinoma	Liver
220	KRAS G12D; STK11 L286R; TP53 F212_ V217delinsL	3.9	Not detected	3.77	Pancreatobiliary adenocarcinoma	Liver
221	TP53 V173L, Amplification KRAS, Amplification CDK6	2.3	Not detected	1.14	Pancreatobiliary adenocarcinoma	Peritoneum
222	CDKN2A L104Rfs*, TP53 R273H	3.13	Not detected	0.88	Pancreatobiliary adenocarcinoma	Liver
223	CDKN2A Y44*, KRAS G12D, SMAD4 Q450fs, TP53 L257R	2.4	Indeterminate	0	Pancreatobiliary adenocarcinoma	Pleura
224	KRAS Q61H	3.16	Not detected	2.25	Pancreatobiliary adenocarcinoma	Peritoneum
225	CDH1 T539fs, CDH1 c.1320+2T>G, Amplification MET	11.85	Not detected	1.98	Gastric adenocarcinoma	Stomach

continue...

...Continuation

Table 4S. Characteristics of all samples with oncogenic/likely oncogenic variants

ID	Oncogenic/ Likely oncogenic variants	TMB (mut/Mb)	MSI status	Percentage of unstable sites	Diagnosis	Sample site
226	TP53 E294fs, CCNE1 Amplification	9.41	Not detected	4	Gastric adenocarcinoma	Esophagogastric junction
227	ERCC2 R487W	2.34	Not detected	0.93	Gastric adenocarcinoma	Stomach
229	TP53 I255N	3.13	Not detected	3.6	Gastric adenocarcinoma	Stomach
230	Amplification ERBB2, ERBB2 D769Y, TP53 R248Q	9.4	Not detected	1.92	Gastric adenocarcinoma	Stomach
231	TP53 R273H, MITF E425K, STAG2 R1186*	0.78	Not detected	0	Adenocarcinoma NOS	Lymph node
232	APC Y935X, TP53 L330fs	Processing	Processing	Processing	Adenocarcinoma NOS	Liver
233	NRAS G12D, TP53 V157F, APC S1465fs, Amplification EGFR, Amplification MYC, Amplification FGF9, Amplification FGF14 (suspicious)	3.13	Not detected	0	Adenocarcinoma NOS	Lymph node
235	BRAF V600E, IDH1 R132C	5.47	Not detected	2.63	Adenocarcinoma NOS	Colon
236	KRAS G12D (subclonal)	3.4	Not detected	4.76	Adenocarcinoma NOS	Omentum
237	PALB2 F638Lfs*, NF1 S1053*, ARID1A K1093Yfs*, TP53 K132Wfs*	9.42	Not detected	1.09	Adenocarcinoma NOS	Peritoneum
238	CDKN2A L78fs, KRAS G12D, GNAS R201H, SMAD4 Y260*, TP53 R273C	3.92	Not detected	0	Adenocarcinoma NOS	Lung
239	MUTYH P143L,SMARCA4 c.3951+1G>T	1.56	Not detected	0.88	Adenosarcoma	Uterus
240	EGFR L858R, TP53 R273L, Amplification MYC	6.4	Not detected	1.37	Adenosquamous carcinoma	Lung
241	KDM6A c.565-2 A >G	10.29	Not detected	1.05	Adenoid cystic carcinoma	Head and neck
242	FH c.556-1G>C	3.95	Not detected	1.19	Adenoid cystic carcinoma	Head and neck
243	SMO D384N (subclonal); PIK3R1 R386*, TP53 R213*, TERT promoter - 146C>T	41.98	Not detected	1.92	Basal cell carcinoma	Eye
245	ARID1A R1461*; KRAS G13D; PTEN Y76*, R130Q	5.48	Not detected	0.99	Endometrial carcinoma	Uterus
246	BRCA2 c.5946delT p.(Ser1982 ArgfsTer22), CDKN2B c.256 G>A p.(Asp86 Asn), MYC Amplification	14.2	Not detected	1.67	Breast carcinoma	Bone
247	PALB2 c.2185_2186insA p.(Pro729 HisfsTer16)	Indeterminate	Indeterminate	1.94	Breast carcinoma	Breast
248	CDH1 c.601_602dupC C p.(Val202 LeufsTer14); PIK3R1 c.1376_1378delA A A p.(Lys459del)	5.49	Not detected	0.86	Breast carcinoma	Liver
249	PIK3CA E545K; ESR1 Y537S	4.7	Not detected	1.68	Breast carcinoma	Liver
250	PIK3CA H1047R; ESR1 D538G; Amplification FGFR1	3.1	Not detected	2.65	Breast carcinoma	Liver
251	ESR1 Y537S; ARID1A F681fs e c.4101+1G>T; Amplification CCND1	4.7	Not detected	1.67	Breast carcinoma	Liver
252	Amplification ERBB2, TP53 Q317*, PIK3R1 N453dup	4.69	Not detected	0	Breast carcinoma	Lung
253	CDKN2A S12*, AKT1 E17K, TP53 G245D, Amplification MYC	7.86	Not detected	0	Breast carcinoma	Lymph node
254	TP53 c.920-1G>A, RB1 R418fs, CIC P1116fs, PIK3R1 D440_E443del, Amplification RET, Amplification KRAS	12.5	Not detected	4.55	Breast carcinoma	Breast
255	TP53 Y234C	2.38	Not detected	3.41	Breast carcinoma	Lymph node
256	ERBB2 Y772_A775dup, CDKN2A H83Y, CDH1 R784Lfs	3.91	Not detected	0.85	Breast carcinoma	Liver
257	Amplification FGFR1, TP53 P301fs, Amplification AR	10.9	Not detected	0	Breast carcinoma	Liver
258	TP53 D281H, Amplification MYC	5.88	Indeterminate	0	Breast carcinoma	Breast
259	PIK3CA H1047R , IDH1 R132C, BCORL1 R784*	3.14	Not detected	0	Breast carcinoma	Breast
260	KRAS c.35 G>A p.(Gly12 Asp); TP53 c.375+1 G>T; SMAD4 c.1529 G>A p.(Gly510 Glu)	1.56	Not detected	3.06	Ovary carcinoma	Vein

continue...

...Continuation

Table 4S. Characteristics of all samples with oncogenic/likely oncogenic variants

ID	Oncogenic/ Likely oncogenic variants	TMB (mut/Mb)	MSI status	Percentage of unstable sites	Diagnosis	Sample site
262	BRCA1 c.4358-66_4364del; TP53 V173L; Amplification MYCL	1.6	Not detected	0.93	Ovary carcinoma	Ovary
263	TP53 A74fs	3.1	Not detected	0.85	Ovary carcinoma	Rectosigmoid
264	TP53 c.646 G>A p.(Val216 Met); ESR1-AKAP12 Fusion	1.57	Not detected	0	Ovary carcinoma	Ovary
265	PIK3CA c.3140 A >G p.(His1047Arg); TP53 c.743 G>A p.(A rg248 Gln), c.839 G>A p.(A rg280 Lys); ARID1A c.488_495delC C G C C G C G p.(A la163 GlyfsTer234); B2M c.280 G>T p.(Glu94 Ter); Fusion NOTCH2-U2AF2	14.1	Not detected	3.42	Ovary carcinoma	Pelvis
266	TP53 K132R	2.3	Not detected	2.6	Ovary carcinoma	Ovary
268	PIK3CA R115L, NRAS Q61H, TP53 R175H	3.15	Not detected	0	Ovary carcinoma	Uterus
270	TP53 V157F; RB1 Q257*	17.98	Not detected	0.86	Small cell lung carcinoma	Lung
271	BARD1 c.365-2A>T, PTEN c.1027-2A>T, TP53 R158L, Amplification MYC	6.28	Not detected	2.61	Small cell lung carcinoma	Lung
272	Fusion TMPRSS2::ERG, AR-V7, Amplification AR, TP53 M133fs	Processing	Processing	Processing	Prostate carcinoma	Lymph node
273	TERT promoter -124C>T	1.6	Indeterminate	0	Thyroid carcinoma	Thyroid
274	NRAS Q61R; TERT promoter -124C>T	1.58	Not detected	1.01	Thyroid carcinoma	Bone
275	KRAS G12V;TP53 E285K;TERT promoter - 124C>T	2.3	Not detected	1.68	Thyroid carcinoma	Thyroid
276	NRAS Q61K, MEN1 I85fs, TERT promoter - 124C>T	2.34	Not detected	2.59	Thyroid carcinoma	Thyroid
277	RET M918T	0	Not detected	1.19	Thyroid carcinoma	Thyroid
278	KRAS G12V, PIK3CA E545K, TERT c.1-124:C>T	12.5	Not detected	2.04	Thyroid carcinoma	Head and neck
279	BLM Y736fs	1.58	Not detected	0	Thyroid carcinoma	Lymph node
280	BRCA2 R2659S; TP53 R273H	14.1	Not detected	1.15	Gallbladder carcinoma	Gallbladder
281	EGFR S768_D770dup, CDKN2A c.151- 1G>C, TP53 E180*, Amplification FGFR1, ATM G2765V	8.62	Not detected	0	Squamous cell carcinoma of the head and neck	Lymph node
282	PALB2 T799fs, TP53 R283P, Amplification CCND1, Amplification CDK6, Amplification MYC	1.59	Not detected	5.17	Squamous cell carcinoma of the head and neck	Lymph node
283	STK11 c.598- 13_606del, Amplification CCND,Amplification FGFR1,Amplification FGF3	3.13	Not detected	0	Squamous carcinoma of the anal canal	Lymph node
284	KDM6A, Amplification PIK3CA N891fs	7.85	Not detected	0	Squamous carcinoma of the anal canal	Rectum
286	PIK3CA E545K	1.56	Not detected	0	Squamous cell carcinoma of the lung	Lung
287	CDKN2A c.116delA p.(Asn39Thrfs*14), ARID1A c.971dupG p.(Ala325Argfs*75), TP53 c.820G>T p.(Val274Phe); Amplification PIK3CA	10.19	Not detected	0	Squamous cell carcinoma of the lung	Thoracic wall
288	TP53 V272L	8.6	Not detected	1.74	Squamous cell carcinoma of the lung	Lung
289	KRAS G12C, STK11 P203Rfs*, TP53 V173L	5.49	Not detected	3.41	Squamous cell carcinoma of the lung	Lung
290	Amplification MYC (suspicious)	2.34	Not detected	0	Squamous cell carcinoma of the lung	Liver
291	TP53 R248Q; NRG1 Amplification	9.4	Not detected	0	Gastric carcinoma	Stomach
292	CTNNB1 S37C, TP53 R248W, SMAD4 R361H, Amplification CCND1	5.5	Not detected	0	Neuroendocrine carcinoma NOS	Abdominal wall
293	TP53 R213*, TP53 R342*, RB1 G449E, BAP1 Q456*, APC I1307K	62.34	Not detected	3.96	Neuroendocrine carcinoma NOS	Head and neck
294	RB1 c.380+2T>C, MUTYH c.494A>G:p.(Tyr165Cys)	3.13	Not detected	3.42	Neuroendocrine carcinoma NOS	Liver

continue...

...Continuation

Table 4S. Characteristics of all samples with oncogenic/likely oncogenic variants

ID	Oncogenic/ Likely oncogenic variants	TMB (mut/Mb)	MSI status	Percentage of unstable sites	Diagnosis	Sample site
295	KRAS c.35 G>C p.(Gly12Ala); APC c.4660 G>T p.(Glu1554 Ter); TP53 c.785 G>T p.(Gly262 Val); RB1 c.2490-4_2496del	3.93	Not detected	3.77	Neuroendocrine carcinoma NOS	Colon
296	ERCC2 Q662*, PTEN G129E, TP53 E224*, PIK3R1 N453dup	4.7	Not detected	6.98	Neuroendocrine carcinoma NOS	Head and neck
297	CDKN2A D84Y, RB1 G310*, TP53 R273S	16.41	Not detected	3.31	Neuroendocrine carcinoma NOS	Lung
298	TP53 A159V, RB1 K289fs	5.49	Not detected	2.27	Neuroendocrine carcinoma NOS	Liver
299	CDKN2A E61*, TP53 Q192*, SDHA D223fs, SMARCA4 c.1245_1245+1delGGinsTT, Amplification PIK3CA, Amplification MYC	6.2	Not detected	1.82	Neuroendocrine carcinoma NOS	Thoracic wall
300	TP53 W91*, PTEN N334fs, AR S760Y, Fusion ATAD2-AR	3.1	Not detected	0	Neuroendocrine carcinoma NOS	Meninges
301	TP53 G226fs, TP53 R342*, Fusion TMPRSS2-ERG, Amplification CCNE1	6.3	Not detected	2.65	Neuroendocrine carcinoma NOS	Lymph node
302	KRAS G12R, Amplification CCNE1, Amplification ALK, Amplification AKT2	8.6	Not detected	4.95	Neuroendocrine carcinoma NOS	Liver
303	KRAS c.35 G>T p.(Gly12 Val); BRIP1 c.3167 C >G p.(Ser1056 Ter); MEN1 c.1200+1_1200+31del, c.788delA p.(Gln263 A rgfs*23); MUTYH c.1145 G>A p.(Gly382 A sp)	1.57	Not detected	1.92	Neuroendocrine carcinoma NOS	Liver
304	ATRX M828*, EPHA7 R895*	4.7	Not detected	1.41	Neuroendocrine carcinoma NOS	Pancreas
305	BAP1 Y223fs, NF2 K130fs, TP53 C135G	3.92	Not detected	0.91	Renal carcinoma	Lung
306	FANCL T367fs, SMAD4 G352R	3.93	Not detected	2.38	Renal carcinoma	Pleura
308	CDKN2A R80*	7.07	Not detected	0	Renal carcinoma	Lymph node
309	MET H1094Y, B2M I27fs*30	4.59	Indeterminate	0	Renal carcinoma	Kidney
310	BRCA1 F107fs, ARID1A T1376fs, APCI1307Kc	2.35	Not detected	0	Renal carcinoma	Peritoneum
311	TP53 F341fs, ARID1BS1262fs	3.13	Not detected	0	Renal carcinoma	Lymph node
313	CDKN2A c.322 G>C p.(A sp108 His); NF1 c.4078 C >T p.(Gln1360 Ter); TP53 c.818 G>T p.(A rg273 Leu), c.1010 G>A p.(A rg337 His); SMARCA4 c.1345 G>T p.(Glu449 Ter); STK11 c.822dupC p.(A sp277 A rgfs*8); KEAP1 c.1525 G>T p.(Gly509 Trp)	21.15	Not detected	0	Carcinoma NOS	Meninges
314	VHL c.34 G>T p.(Glu12 Ter), Amplification AKT2	3.93	Not detected	0	Carcinoma NOS	Lymph node
315	KRAS Q61H; STK11 S271fs; PBRM1 c.1302-1G>A	5.5	Not detected	1.43	Carcinoma NOS	Pelvis
316	KRAS G12D; STK11 G215fs, C278fs	6.3	Not detected	1.79	Carcinoma NOS	Lung
317	KRAS c.34 G>T p.(Gly12 Cys); TP53 c.713 G>T p.(Cys238 Phe)	26.63	Not detected	1.01	Carcinoma NOS	Lymph node
318	NRAS Q61R, BAP1 Q28Rfs*, TP53 Q136P	3.92	Not detected	2.54	Carcinoma NOS	Lung
319	BRCA1 E303fs, CDKN2A H66fs e c.151-1G>A, TP53 c.993+1G>A, TERT promoter - 146C>T	5.47	Not detected	1.71	Carcinoma NOS	Lung
321	CDKN2A D116fs	0.78	Not detected	0	Carcinoma NOS	Omentum
322	CTNNB1 S37F, CUL3 R59* , PIK3C2G Q391*	36.84	Not detected	0	Carcinoma NOS	Lymph node
323	Deletion CDKN2A/B, ARID1A P1903fs, TP53 D61fs, Amplification MYCN	10.98	Not detected	0	Carcinoma NOS	Liver
324	BRAF V600E, TP53 T150fs, CTCF I17fs	6.32	Not detected	0	Carcinoma NOS	Prostate
325	TP53 C238F, RB1 G100*, Amplification CCNE1, Amplification MYCL	6.28	Not detected	1.3	Carcinoma NOS	Esophagus
326	KRAS G13D, STK11 E145*, SMARCA4 W942*, TP53 S241F	10.2	Not detected	1.75	Carcinoma NOS	Lymph node
327	APC I1307K	3.14	Not detected	0	Carcinoma NOS	Ovary
328	CDKN2A H83Y, KRAS G12D, TP53 R213*, SMAD4 G386V	0.78	Not detected	0	Carcinoma NOS	Lung

continue...

...Continuation

Table 4S. Characteristics of all samples with oncogenic/likely oncogenic variants

ID	Oncogenic/ Likely oncogenic variants	TMB (mut/Mb)	MSI status	Percentage of unstable sites	Diagnosis	Sample site
330	NF1 N1652Tfs, PTEN T319*, MLH1 E37K, FANCA c.3766- 2A>G, PBRM1 N258Kfs*	30.6	Detected	28.72	Carcinoma NOS	Soft tissue
332	BRAF V600E, ARID1A G313fs, ARID2 Q1575*	5.5	Not detected	0	Carcinoma NOS	Liver
333	KEAP1 c.1336 G>T p.(Glu446 Ter); CTNNB1 c.101 G>T p.(Gly34 Val); TP53 c.880 G>T p.(Glu294 Ter); SMARCA4 c.1745delA p.(Lys582 A rgfsTer31)	17.3	Not detected	1.72	Carcinoma NOS	Lymph node
334	NF2 c.570_599+11del	4.68	Not detected	2.63	Carcinoma NOS	Pleura
335	Fusion EWSR1-WT1	4.7	Not detected	1.65	Carcinoma NOS	Peritoneum
336	TP53 Y103*; Amplification MYC; Amplification CCNE1	3.9	Not detected	0.88	Urothelial carcinoma	Pleura
337	TERT c.-124 C >T; TP53 c.396 G>C p.(Lys132 A sn), c.548 C >G p.(Ser183 Ter); RB1 c.963 C >A p.(Tyr321 Ter)	11.72	Not detected	4.59	Urothelial carcinoma	Bladder
338	BRCA1: c.4621 G>T (p.Glu1541 Ter), TERT c.-124 C >T, TP53 c.637 C >T (p.A rg213 Ter), RB1 c.242dupT (p.Ser82 Phefs*28), ARID1A c.1435 C >T (p.Gln479 Ter)	16.46	Not detected	1.92	Urothelial carcinoma	Bladder
339	TERT c..-124 C >T, ARID1A c.31_56del p.(Ser11Alafs*91), RB1 c.751C>T p.(Arg251Ter)	9.4	Not detected	1.43	Urothelial carcinoma	Kidney
340	KDM6A c.3592_3601delinsA p.(Glu1198_ Tyr1201delinsA sn); TERT c.-124 C >T; TP53 c.1006 G>T p.(Glu336 Ter); RAC1 c.85 C >A p.(Pro29 Thr)	8.62	Not detected	2.63	Urothelial carcinoma	Bladder
341	FGFR3 c.746 C >G p.(Ser249 Cys) e Amplification; ERBB2 c.929 C >T p.(Ser310 Phe); PIK3CA c.331 A >G p.(Lys111 Glu); KDM6A c.736_739delT TA C p.(Leu246 A snfsTer3); TERT c.-124 C >T; CDKN1B c.318_321delCCAG p.(Ser106 A rgfsTer12); KEAP1 c.1258dupG p.(Val420 GlyfsTer25); MDM2 Amplification	23.5	Not detected	2.86	Urothelial carcinoma	Bladder
342	TERT promoter -124C>T; RB1 c.1960+1G>A	11	Not detected	2.08	Urothelial carcinoma	Bladder
343	TSC1 Y185X; TP53 I195T; Amplification CCND1; Amplification RICTOR	3.1	Not detected	1.22	Urothelial carcinoma	Bladder
344	TERT promoter c.1-124:C>T	10.2	Not detected	3.45	Urothelial carcinoma	Liver
345	TSC1 R500*; CDKN2A T18_A19del; TP53 c.97-1G>A; SMARCA4 c.2275-1G>C; TERT promoter -124C>T	17.2	Not detected	0	Urothelial carcinoma	Lymph node
346	TERT promoter - 124C>T; ARID1A S645*; RB1 Q62*; TP53 R273H	10.9	Not detected	3.45	Urothelial carcinoma	Lymph node
347	ERBB2 L755S; ERBB2 V842I;BRCA1 K654fs; CHEK1 T226fs; NF1 R461*; ARID1A D1850fs; ATR I774fs; MSH2 Q824*; MSH6 F1088fs	69	Detected	49.15	Urothelial carcinoma	Bladder
348	PIK3CA E545K, BRAF L597Q, ATR c.5739- 1G>C, TP53 K132N	9.42	Not detected	3.75	Urothelial carcinoma	Bladder
349	NRAS G13R, KDM6A c.385- 2A>G, ARID1A Q611	5.47	Not detected	2.48	Urothelial carcinoma	Lymph node
350	ARID1A G285*, TERT promoter -124C>T, Amplification CCND1	3.92	Not detected	1.27	Urothelial carcinoma	Lymph node
351	ERCC2 T484M; BARD1 E59fs; ATM S214fs; KDM6 N634fs; TERT promoter -124C>T; BAP1 Q36*, c.1251-1G>A; CDH1 T38fs	23.4	Not detected	3.45	Urothelial carcinoma	Retroperitoneum
352	KDM6A Q677*, ARID1A Q386*, SUFU A340fs, TP53 R333fs, TERT promoter c.-146C>T, Amplification AKT2, Amplification MYCN	27.5	Not detected	0	Urothelial carcinoma	Bladder
353	Amplification MDM2; Amplification MYC	0.78	Not detected	7.45	Urothelial carcinoma	Right kidney

continue...

...Continuation

Table 4S. Characteristics of all samples with oncogenic/likely oncogenic variants

ID	Oncogenic/ Likely oncogenic variants	TMB (mut/Mb)	MSI status	Percentage of unstable sites	Diagnosis	Sample site
354	PIK3CA c.3140 A >G p.(His1047 Arg); CHEK1 c.676dupA (p.Thr226 A snfsTer19); KRAS c.436 G>A p.(A la146 Thr); PTEN c.81 T>A p.(Tyr27 Ter), c.976_990del p.(Lys330_A sn334del); CIC c.3743delC p.(Pro1248 HisfsTer54), c.4586delC p.(Pro1529 LeufsTer91), c.4790delC p.(Pro1597 HisfsTer23); STK11 c.842delC p.(Pro281 A rgfsTer6); ARID1A c.4563delC p.(A la1522 ProfsTer5), c.2434 C >T p.(Gln812 Ter); MSH6 c.3261dupC p.(Phe1088 LeufsTer5); SUFU c.71dupC p.(A la25 GlyfsTer23)	61.3	Detected	66.67	Carcinosarcoma	Uterus
355	PIK3CA E453K; Amplification FGFR3; Amplification MYC; TP53 R282G; Fusion STAT5-BBRCA1	6.2	Not detected	4.21	Carcinosarcoma	Lymph node
356	IDH1 c.394 C >T p.(A rg132 Cys)	7.08	Not detected	0.98	Cholangiocarcinoma	Liver
357	ATM S681fs; KRAS G13D; ARID1A S11fs; Amplification CCND1	11	Not detected	0	Cholangiocarcinoma	Liver
358	NRAS Q61R, BAP1 c.67+1G>A	0.78	Not detected	0.85	Cholangiocarcinoma	Liver
359	BRAF V600E	2.3	Not detected	1.16	Cholangiocarcinoma	Liver
360	IDH1 R132L; NF2 R196*	2.3	Not detected	2.73	Cholangiocarcinoma	Liver
362	CDKN2A M52*, KRAS G12D, TP53 c.993+2T>A	3.14	Not detected	0	Cholangiocarcinoma	Liver
363	KRAS G12S	2.36	Not detected	0	Cholangiocarcinoma	Liver
364	PBRM1 R1095*, TP53 L194R, D281H	3.14	Not detected	0	Cholangiocarcinoma	Peritoneum
365	KRAS G12C (subclonal)	4.73	Not detected	0	Cholangiocarcinoma	Peritoneal/adrenal lesion (right)
366	IDH1 132, BRAF G464V, ARID1A S617Qfs*2	4.7	Not detected	0	Cholangiocarcinoma	Liver
367	BRAFV600E, TP53Y220,TERT c.- 124C>T	3.92	Not detected	0	Cholangiocarcinoma	Liver
369	TP53 R156H	0.78	Not detected	1.39	Ganglioneuroblastoma	Mediastinum
370	KIT c.1504_1509dup p.(Ala502_Tyr503dup), c.2459A>G p.(Asp820Gly)	5.61	Not detected	1.96	GIST	Small intestine
371	KIT A502_Y503dup	0	Not detected	0	GIST	Rectum
372	Fusion KIAA1549::BRAF	2.41	Indeterminate	0	Glioma	Spinal cord
373	Fusion KIAA1549::BRAF	0.78	Not detected	0	Glioma	Brain
375	EGFR c.866 C >T p.(A la289 Val), c.1787 C >T p.(Pro596 Leu), Amplification; PTEN c.548dupA p.(A sn184 GlufsTer6); FLCN c.715 C >T p.(A rg239 Cys)	4.7	Not detected	0.85	Glioma	Brain
376	EGFR A289T; PIK3R1 G376R; RB1 E464*	3.1	Not detected	2.54	Glioma	Brain
377	EGFR A289V; EGFR Ampplificação;TERT c.-124C>T	5.4	Not detected	4.63	Glioma	Brain
378	PTEN N12D, RB1 R254fs, TP53 R248W	8.6	Not detected	1.83	Glioma	Brain
379	EGFR A289V, Amplification; CDKN2A V59fs; TERT promoter c.-124C>T	1.58	Not detected	7.69	Glioma	Brain
380	PTEN R130Q, TERT promoter -124C>T	3.9	Not detected	2.15	Glioma	Brain
381	EGFR vIII; EGFR c.323 G>A p.(Arg108 Lys); EGFR Amplification; Fusion SEC 61G-EGFR; TERT c.-124 C >T; TP53 c.817 C >T p.(A rg273 Cys)	1.58	Not detected	2.35	Glioma	Brain
382	Fusion KIAA1549- BRAF	0.8	Not detected	1.72	Glioma	Brain
383	ARID1A Q449_S2285delinsH; TP53 F212fs, R248W; TERT c.-124C>T; Amplification PDGFRA; Amplification KIT	1.5	Not detected	2.38	Glioma	Brain
384	IDH2 R172T, TP53 R273C, ATRX K897fs	4.75	Not detected	2.53	Glioma	Brain

continue...

...Continuation

Table 4S. Characteristics of all samples with oncogenic/likely oncogenic variants

ID	Oncogenic/ Likely oncogenic variants	TMB (mut/Mb)	MSI status	Percentage of unstable sites	Diagnosis	Sample site
385	PIK3R1 R358*; TP53 R248Q, *394Ifs*26; H3-3A G35R; PTEN R130Q; ATRX V1678del	7.03	Not detected	0.88	Glioma	Brain
386	Fusion KIAA1549- BRAF	3.92	Not detected	2.65	Glioma	Brain
387	Fusion KIAA1549::BRAF	0.78	Not detected	0	Glioma	Brain
388	PTEN Y27C, Amplification CDK4, Amplification MDM4, TERT promoter - 146	0.78	Not detected	1.19	Glioma	Brain
389	IDH1 R132H, CIC Q1110*, FUBP1 N369fs, TERT promoter 124C>T	3.14	Not detected	0	Glioma	Brain
390	R132H, ATRX I457fs, TP53 R248W, PIK3R1 E439del	4.72	Not detected	0	Glioma	Brain
391	IDH1 R132H, Amplification KRAS, Amplification FGF6, Amplification FGF23	4.7	Not detected	0	Glioma	Brain
392	BRAF N486_T491delinsK, Deletion CDKN2A/B	4.34	Not detected	0	Glioma	Brain
393	BRAF V600E	0	Not detected	0	Glioma	Brain
395	H3-3AK28M, TP53E258G	1.57	Not detected	0	Glioma	Cerebellum
396	TP53 I195T, TP53 R175H, PIK3R1 R301*, Amplification CDK4	1.56	Not detected	0	Glioma	Brain
397	IDH1 R132H, TERT promoter -146C>T, TP53 Y220C(subclonal)	1.56	Not detected	0	Glioma	Brain
398	PIK3CA H1047R, H3C2 K28M, ACVR1 R258G	3.14	Not detected	0	Glioma	Brain
399	EGFR T790M, Amplification EGFR,TERT c.-124C>T (promoter), Deletion CDKN2A/B	Processing	Processing	Processing	Glioma	Brain
400	IDH1 R132H, ATRX K1583f, TP53M246V	0.78	Not detected	0	Glioma	Brain
401	Fusion LHFPL3-BRAF	0	Not detected	1.19	Glioma	Brain
402	Fusion FGFR2- SHTN1; TP53 G245S; APC I1307K	0.79	Not detected	5.49	Glioma	Brain
404	NF1 Q1822*, BLM W1288*, TERT promoter -124C>T	81.7	Not detected	5.71	Melanoma	Skin
405	BRAF V600K, TERT promoter - 146C>T	20.31	Not detected	1.75	Melanoma	Lymph node
406	NRAS Q61K	4.74	Not detected	7.84	Melanoma	Skin
407	NRAS Q61R, Amplification; TERT promoter -124C>T	10.97	Not detected	4.5	Melanoma	Lymph node
408	NF1 R440*, CDKN2A P114L, TERT promoter -146C>T, TP53 R342*, APC Q445*	82.2	Not detected	2.17	Melanoma	Liver
409	MAP2K1 E203K, BARD1 K127*, ARID1A Q553fs, SDHD R122*,TERT promoter -146C>T	25.1	Not detected	1.02	Melanoma	Skin
410	SF3B1 R625H, KMT2D K866fs	2.34	Not detected	0	Melanoma	Ocular conjunctiva
411	NRAS Q61R, TERT promoter -124C>T	10.97	Not detected	0	Melanoma	Skin
412	GNAQ Q209P, SF3B1 R625H	1.56	Not detected	0	Melanoma	Orbit
413	NF2 c.448- 1G>T	1.56	Not detected	0	Meningioma	Cerebellum
414	BRCA2 N1473fs; BAP1 c.1251-2A>T	2.3	Not detected	5.98	Mesothelioma	Lung
415	NF2 R196fs	1.6	Not detected	3.6	Mesothelioma	Peritoneum
416	BAP1 c.660-2A>C	3.9	Not detected	0.85	Mesothelioma	Peritoneum
417	KRAS G12C, Amplification MET, TP53 K132R	10.94	Not detected	2.5	Mucoepidermoid	Lung
419	IDH1 R132H, ATRXc.6699+1G>A, TP53 I232fs	6.29	Not detected	0	NA	Brain
421	MAP2K1 E102_I103del	0.79	Not detected	0	Histiocytic neoplasm	Lymph node
422	NRAS Q61R	0	Not detected	0	Histiocytic neoplasm	Soft tissue
426	ATM Q2433*, NRAS Q61H, ATR Q1561*, FUBP1 R344*, TERT promoter c.- 146 C>T, RAC1 P29S	57.18	Not detected	4.5	Sarcoma	Lymph node
427	Amplification CDK4; Amplification MDM2	5.5	Not detected	6.82	Sarcoma	Retroperitoneum

continue...

...Continuation
Table 4S. Characteristics of all samples with oncogenic/likely oncogenic variants

ID	Oncogenic/ Likely oncogenic variants	TMB (mut/Mb)	MSI status	Percentage of unstable sites	Diagnosis	Sample site
428	MEN1 c.928-1 G>A, MSH2 c.1165C >T p.(Arg389Ter), MUTYH c.1145G>A p.(Gly382Asp), RB1 c.1959dupA p.(Val654 Serfs*14), RAD50 c.2801dupA p.(A sn934 Lysfs*10)	24.37	Detected	22.22	Sarcoma	Thoracic wall
429	Amplification CDK4, Amplification MDM2	1.57	Not detected	1.68	Sarcoma	Retroperitoneum
431	TP53 c.375+1G>A, RB1 c.540-2A>T	5.6	Not detected	2.86	Sarcoma	Soft tissue
432	PTCH1 N97fs; BRCA2 T3033fs; NF1 E1929fs; TP53 R273C; MSH6 G1105fs; ATR c.3451-1G>T	13.4	Not detected	12.63	Sarcoma	Retroperitoneum
433	ATRX E669fs; TP53 c.782+1G>A	4.7	Not detected	4.76	Sarcoma	Uterus
434	NF1 R2258X	10.1	Not detected	1.94	Sarcoma	Retroperitoneum
436	IDH1 R132C	3.13	Not detected	3.23	Sarcoma	Pelvis
439	KDM6A R519*, TP53 R337H, Amplification RICTOR, Amplification MDM2, Amplification CCNE1	3.17	Not detected	2.5	Sarcoma	Soft tissue
440	RB1 L486fs	3.1	Not detected	3.14	Sarcoma	Soft tissue
441	PTPN11 E76A, KDM6A c.3878+2insTGCCTTCCCCT, TP53 P152L	7.89	Not detected	1.94	Sarcoma	Small intestine
442	Amplification CDK4, Amplification MDM2, Amplification ESR1	3.93	Not detected	0	Sarcoma	Soft tissue
443	Fusion LMNA-NTRK1, MUTYH c.1145 G>A p.(Gly382 A sp)	3.93	Not detected	2.7	Sarcoma	Lung
444	Fusion PAX3-FOXO1	1.57	Not detected	0	Sarcoma	Uterus
446	TP53 R158G, Fusion RP11- 35609.1::ETV1	0.82	Not detected	0	Sarcoma	Uterus
448	Fusion PAX3::NCOA2	0	Not detected	0	Sarcoma	Head and neck
449	TP53 I195N, RB1 S618*, Deletion CDKN2A/B	3.13	Not detected	0	Sarcoma	Soft tissue
450	Amplification MYC, Amplification CCNE1, Amplification FGF2	4.7	Not detected	0	Sarcoma	Bone
451	TP53 T230Afs, Amplification CDK6	7.06	Not detected	0	Sarcoma	Soft tissue
452	Amplification KRAS; TP53 Y220C	7.8	Not detected	1.8	Germ cell tumor	Lung
454	TP53 c.96+1G>C, TP53 Y234*	1.57	Not detected	0	Solitary fibrous tumor	Liver

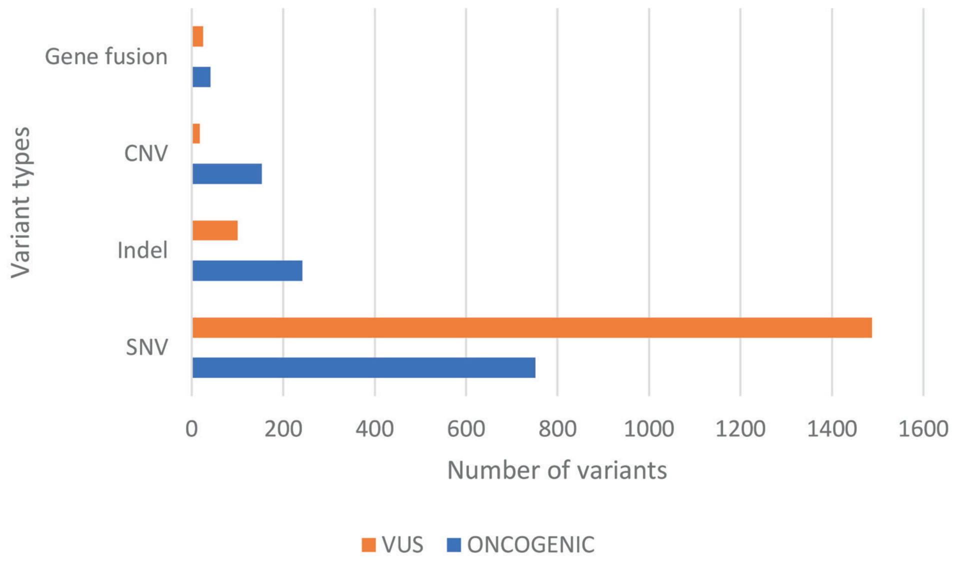

CNV: copy number variants; VUS: variants of uncertain significance.

Figure 3S. Frequency of variant types in the samples subjected to comprehensive genomic profiling

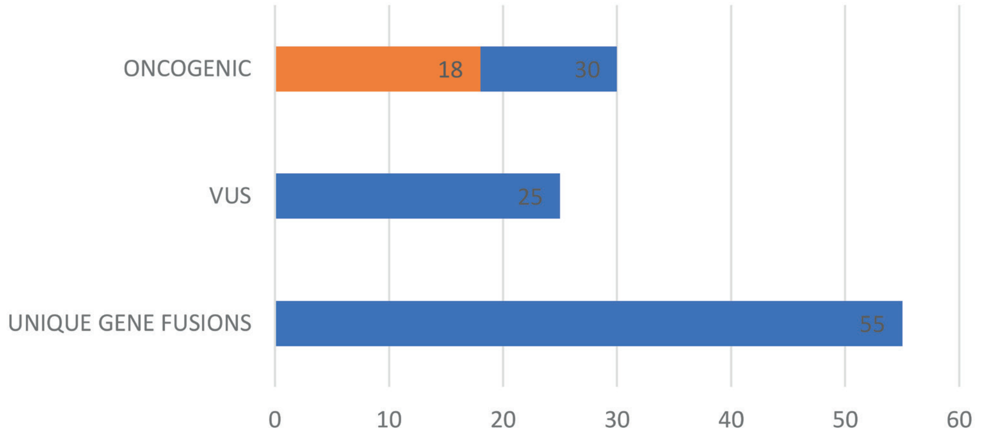

VUS: variants of uncertain significance.

Figure 4S. Gene fusions identified in the cases subjected to comprehensive genomic profiling
